# A functional SNP regulates E-cadherin expression by dynamically remodeling the 3D structure of a promoter-associated non-coding RNA transcript

**DOI:** 10.1093/nar/gkac875

**Published:** 2022-10-16

**Authors:** Shrikant Sharma, Giuseppina Pisignano, Jessica Merulla, Carlo V Catapano, Gabriele Varani

**Affiliations:** Department of Chemistry, University of Washington, Seattle, WA 98195-1700, USA; Department of Biology and Biochemistry, University of Bath, Bath BA2 7AY, UK; Institute of Oncology Research (IOR), Università della Svizzera italiana (USI), 6500 Bellinzona, Switzerland; Institute of Oncology Research (IOR), Università della Svizzera italiana (USI), 6500 Bellinzona, Switzerland; Department of Chemistry, University of Washington, Seattle, WA 98195-1700, USA

## Abstract

Transcription of E-cadherin, a tumor suppressor that plays critical roles in cell adhesion and the epithelial-mesenchymal transition, is regulated by a promoter-associated non-coding RNA (paRNA). The sense-oriented paRNA (S-paRNA) includes a functional C/A single nucleotide polymorphism (SNP rs16260). The A-allele leads to decreased transcriptional activity and increased prostate cancer risk. The polymorphic site is known to affect binding of a microRNA-guided Argonaute 1 (AGO1) complex and recruitment of chromatin-modifying enzymes to silence the promoter. Yet the SNP is distant from the microRNA-AGO1 binding domain in both primary sequence and secondary structure, raising the question of how regulation occurs. Here we report the 3D NMR structure of the 104-nucleotide domain of the S-paRNA that encompasses the SNP and the microRNA-binding site. We show that the A to C change alters the locally dynamic and metastable structure of the S-paRNA, revealing how the single nucleotide mutation regulates the E-cadherin promoter through its effect on the non-coding RNA structure.

## INTRODUCTION

Classical cadherins, such as E-cadherin, are transmembrane glycoprotein components of adherens junctions which promote intercellular communication (1,2). The E-cadherin gene (CDH1) generates a 120 kDa protein (3,4) whose cytoplasmic domain links various catenins to the actin cytoskeleton and facilitates downstream signaling through multiple pathways, including Wnt and TGF-β (5). Dysfunction of E-cadherin is linked to invasiveness and advanced tumor progression in many epithelial cancers, and reduced E-cadherin expression is a hallmark of the epithelial-mesenchymal transition (EMT), while its inhibition provokes detachment and invasion of cancer cells (6,7). Thus, E-cadherin acts as a tumor suppressor whose transcriptional deregulation promotes carcinogenesis in epithelial tissues (8).

**Figure 1.**
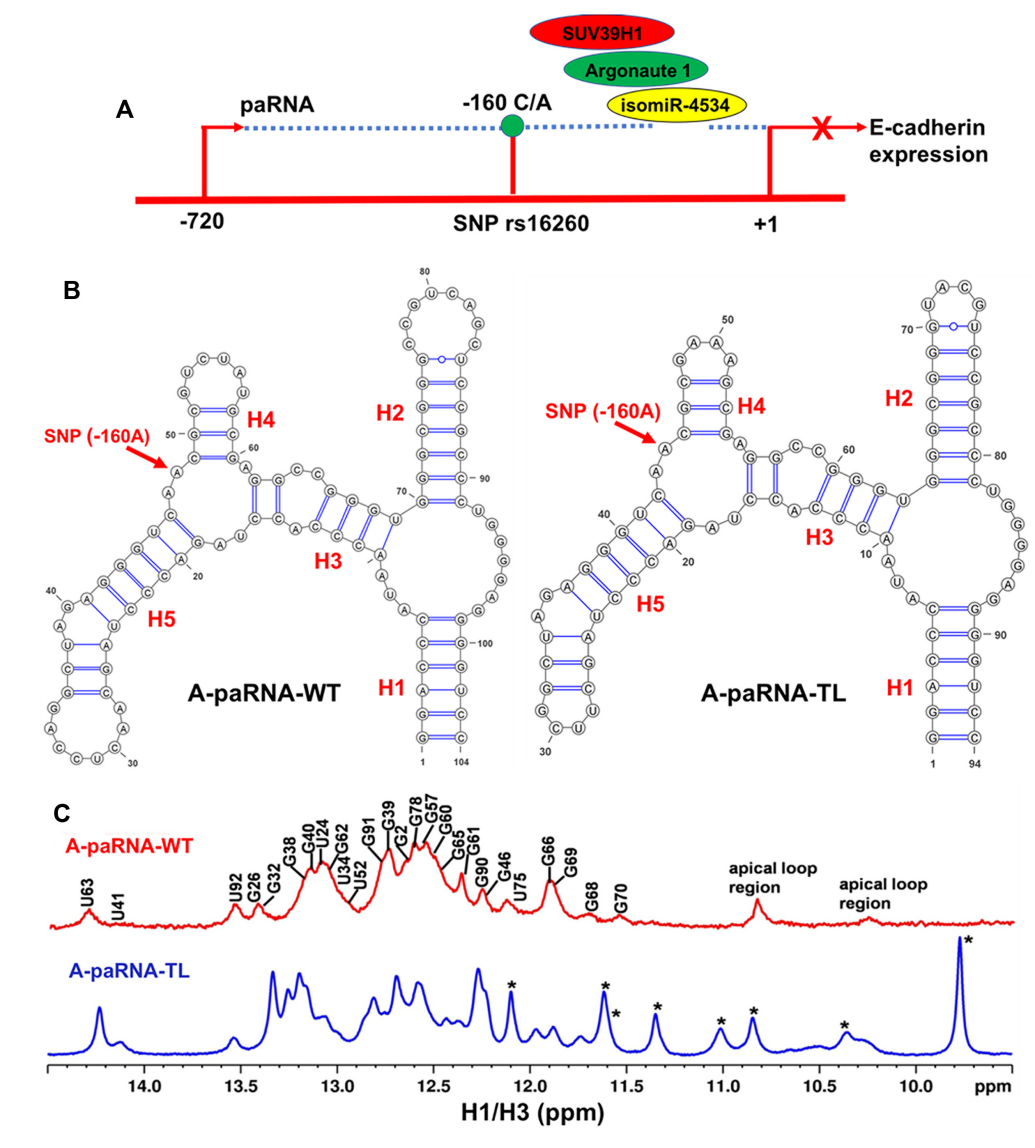
(**A**) Schematic diagram of the impact of SNP rs16260 on transcriptional regulation of E-cadherin. (**B**) Secondary structure of the domain of the CDH1 paRNA that regulates E-cadherin transcription, named A-paRNA-WT, and of its variant A-paRNA-TL, where tetraloops were introduced to stabilize the RNA structure and reduce aggregation which caused poor spectral quality. (**C**) Comparison of the 1D imino ^1^H NMR spectra of the two RNAs, recorded at 15°C, with assignments for the A-paRNA-TL, obtained as presented in the text, mapped onto the spectrum of the sequence with wild type loops. The spectra are very similar, indicative of conserved structures; Asterisks identify resonances originating from the UUCG, GAAA and UACG tetraloops, within the A-paRNA-TL construct.

Hypermethylation of the CDH1 promoter has been observed in human breast, prostate, and hepatocellular tumors carrying a wildtype CDH1 gene, leading to reduced expression of E-cadherin (9,10). A C/A polymorphism (SNP rs16260) at position –160 from the transcriptional start site decreases the activity of the CDH1 promoter by about 70% and is linked to increased risk for prostate cancer (11). The C allele more robustly recruits transcription activating factors compared to the A allele (11). We showed previously that silencing of the CDH1 promoter requires the formation of a microRNA (miRNA)-guided Argonaute 1 (AGO1) complex on an independently transcribed sense promoter-associated non-coding transcript (S-paRNA) which encompasses the SNP and recruits the SUV39H1 methyltransferase to induce repressive chromatin modifications (12) (Figure 1A). We demonstrated that the SNP-160 (C/A) influences the ability of a miRNA (isomiR-4534) and AGO1 to interact with the S-paRNA (12). However, it remains mechanistically unclear how the polymorphism affects the miRNA–AGO1 interaction with S-paRNA and transcriptional regulation, since it does not overlap with the miRNA binding site in sequence or secondary structure. The A and C variants differ substantially in their patterns of SHAPE reactivity at and near SNP rs16260, revealing that the secondary structures for the two polymorphic transcripts must be different (12), but why and how the secondary structure would change is also not clear.

We report here the 3D structure of the 90-nucleotide domain within the S-paRNA containing the A- and C-SNPs, as determined by NMR spectroscopy. We show that the S-paRNA folds into a well-defined three-dimensional structure, and that the A→C mutation unfolds a three-way junction where the SNP is located. Propagation of this rearrangement requires the presence of a neighboring flexible internal loop that disrupts the helix that links the three-way junction to the miRNA-AGO1 binding site. When the internal loop is replaced with base pairs, the conformational change disappears and AGO1-binding to the promoter becomes independent of the A or C SNP identity. These observations introduce a new mechanism for regulation of non-coding RNA function, which emphasizes the functional role of RNA structural dynamics and the potential for its modulation by genetic variation. Moreover, since our study suggests that changes in the structure of non-coding RNAs at polymorphic sites or through somatic mutations can affect the epigenetic landscape of cancer promoting genes, these findings also suggest new strategies for therapeutic targeting.

## MATERIALS AND METHODS

### RNA synthesis, purification and NMR sample preparation

All RNA constructs were prepared by *in vitro* transcription using *in house* purified T7 RNA polymerase and synthetic DNA templates (purchased from Integrated DNA Technologies), as reported (13,14). 2′-O-methyl groups were incorporated with the last two residues at the 5′ end of the templates to reduce the addition of untemplated nucleotides at the 5′-end. ′Ultramer′ oligonucleotides were used to prepare RNAs longer than 75 nucleotides. Deuterated RNA samples were prepared in the same way using selective deuterated rNTPs (D-H5, H3′, H4′, H5′ and H5″) and ^13^C/^15^N-labeled samples were prepared using labeled rNTPs (Cambridge Isotope Laboratories) (15,16). The resulting RNAs were purified for NMR studies by gel electrophoresis, electroelution and extensive dialysis, following standard methods (13,14). All NMR experiments were carried out in a final NMR buffer containing 20 mM potassium phosphate (pH 6.0), and 0.01 mM EDTA. For NMR experiments examining non-exchangeable protons, RNA samples were lyophilized and re-dissolved in 99.99% D_2_O. RNA samples used to study exchangeable protons were dissolved instead in 95% H_2_O/5% D_2_O. RNA concentrations used for all NMR experiments were 0.5–1 mM.

Employing a strategy that has been used in several previous RNA studies (15,17), we introduced tetraloops in the A-paRNA-WT sequence, to generate a more stable construct, dubbed A-paRNA-TL, as shown in Figure 1B. Tetraloops were substituted in place of three existing loops, based on the SHAPE-generated secondary structure (12). This is similar to what is often done in RNA x-ray crystallography, where protein binding sites and tertiary contacts are engineered to stabilize crystal contacts in order to facilitate crystallization and improve resolution (18). We reasoned that introducing tetraloops would stabilize the secondary structure and reduce aggregation, and indeed the spectral quality improved considerably.

### NMR experiments

All NMR datasets were recorded on Bruker Avance 800 or/and 600 MHz NMR spectrometers equipped with ^1^H/^13^C/^15^N triple resonance cryogenic probes. Before each experiment, samples were freshly annealed by quick cooling after heating to 90°C. The 1D ^1^H spectra in 95% H_2_O/5% D_2_O were recorded using the excitation sculpting pulse sequence (19). 2D ^1^H–^1^H nuclear Overhauser effect (NOESY) spectra were recorded at both 15°C and 25°C. 2D ^1^H–^1^H NOESY and 2D [^1^H–^15^N] HSQC spectra were recorded in 95% H_2_O/5% D2O in 20 mM phosphate (pH 6.0) and 0.01 mM EDTA, at 25°C and 15°C. In addition, 2D ^1^H–^1^H NOESY, ^1^H–^1^H TOCSY (spin lock 60–80 ms), 2D [^1^H–^13^C] HSQC, and ^1^H–^13^C edited 3D NOESY-HSQC spectra were recorded in 99.9% D_2_O buffer at 25°C. The NOESY spectra were recorded with various mixing times in the range of 100–300 ms to facilitate spectral assignments and quantification of cross peak intensities for structure determination by comparison with peaks corresponding to fixed distances (e.g. H5–H6, H1′–H3′). ^1^H chemical shifts were referenced relative to external sodium 2,2-dimethyl-2-silapentane-5 sulfonate (DSS).

### NMR spectral assignments

In order to determine the structure of the A-paRNA-TL construct containing the A-allele and stabilizing tetraloops, we used a divide-and-conquer approach and divided the RNA into four segments (Supplementary Figure S1, Supplementary Table S1), which overlap to generate the complete structure. We observed many NH peaks in the 1D ^1^H NMR spectrum for the for the full RNA domain and the shorter fragments, revealing a well-folded structure (Supplementary Figure S2). Overlaying the 1D ^1^H and 2D ^1^H–^1^H NOESY NMR spectra of the smaller segments on the spectra of the complete A-paRNA-TL revealed a highly transferable pattern of chemical shifts and NOESY cross-peaks for both the exchangeable and non-exchangeable protons (Supplementary Figures S2–S4). In order to further simplify the spectra, we also prepared deuterated RNA samples (D-H5, H3′, H4′, H5′ and H5″) for the full A-paRNA-TL domain and the smaller fragments shown in Supplementary Figure S5, and recorded 2D ^1^H–^1^H NOESY spectra at different mixing times for these samples as well. Overlaying these simplified, deuterated 2D ^1^H–^1^H NOESY spectra for the smaller domains on those of the full RNA, we again observed considerable similarities in chemical shifts of the ribose sugar and aromatic regions, further supporting the correspondence of the ^1^H chemical shifts between the two constructs (Supplementary Figure S4). Thus, by collecting and comparing different 1D ^1^H, 2D ^1^H–^1^H NOESY and 3D NOESY-HSQC NMR spectra, we were able to transfer chemical shift assignments and NOEs from the smaller RNA sub-domains to the complete A-paRNA-TL domain (Supplementary Figures S2–S4). Spectral assignments of all fragments were facilitated by predicted RNA chemical shifts for helical regions (20) and confirmed using established NOE helical ‘walk’ patterns (13,14) (Supplementary Figures S6–S9). Altogether, the analysis of multiple fragments enabled the bulk of the chemical shift assignments and NMR restraints to be obtained for the structural calculation of the complete A-paRNA-TL domain.

### NMR structural constraints and structure calculations

In order to determine the 3D structure of the A-paRNA-TL, we used the same divide and conquer approach used to assign chemical shifts because it allowed us to obtain a much larger number of distance restraints for structure calculation than would be possible if we had just examined the complete RNA (15,16). The large molecular weight of the full-length construct would lead to prohibitive peak broadening due to fast relaxation of the NMR signal (Supplementary Figure S10). This approach is possible because RNA structure is highly modular (21), and individual stem–loops fold independently of each other; tertiary interactions, if present, are often weak and involve weaker long-range interactions between pre-formed RNA secondary structure motifs that only seldom disrupt the secondary structure (22). After dividing the A-paRNA-TL domain into four partially overlapping smaller segments (Supplementary Figure S1) which, together, generate the complete structure, we obtained the bulk of distance restraints to use for the structure calculation from NOE intensities observed for the smaller domains and by comparing them with those seen for the complete domain. This is possible because, once again, overlaying the 1D ^1^H and 2D ^1^H–^1^H NOESY NMR spectra of the smaller segments on the full A-paRNA-TL spectra revealed a highly transferable pattern of chemical shifts and NOESY cross-peaks for both the exchangeable and non-exchangeable protons (Supplementary Figures S2-S4), as discussed above. This observation strongly suggests that the structure observed in the individual fragments is retained in the complete A-paRNA-TL domain, because chemical shifts are very sensitive to even minor structural changes.

For structure calculations, NOE intensities collected at 100 ms mixing time were binned into ‘strong’ (2.5 ± 0.7 Å), ‘medium’ (3.5 ± 1.5 Å) and ‘weak’ (4.5 ± 2.0 Å) based on peak intensities relative to fixed A-form helical distances (e.g. H5–H6 = 2.5 Å, H3′–H6/H8 = 3.5 Å). For structure calculations, loose (±30°) dihedral angle constraints were introduced for 5 backbone dihedral angles (*α*, *β*, *γ*, *ϵ* and *ζ*). The sugar conformation and population of the major C2′-endo and C3′-endo conformers, which are most common, were estimated from the observed peak intensities in 2D ^1^H–^1^H TOCSY spectra and the angle δ was constrained accordingly. Hydrogen bonds, planarity and dihedral restraints were included for base-paired nucleotides conforming to A-form helical patterns.

Xplor-NIH (23) was used for structure calculations based on a simulated annealing protocol. The target function for refinement included NOE and hydrogen bond distance constraints (force constant = 50 kcal mol^−1^Å^2^) as well as dihedral torsion angle restraints (force constant = 200 kcal mol^−1^ rad^−2^). The simulated annealing procedure started from randomized coordinates and initially subjected the system to high temperature torsion angle dynamics (8000 steps at 3500 K). At first, only NOE and Van Der Waals terms were employed to fold the structure. Initial convergence was established when no NOE violations greater than 0.5 Å remained in the calculated structures. After this initial folding step, base-pair planarity and hydrogen-bonding restraints were examined for unambiguously established base pairs as identified from 2D ^1^H–^1^H NOESY and ^1^H–^15^N HSQC experiments. The bath temperature was gradually cooled from 3500 K to 298 K while introducing the van der Waals terms and incrementally raising the force constants for a number of terms (bond angles, impropers, dihedral angles, NOEs, van der Waals repulsion and ‘RAMA’). Following the final cooling step, the molecules underwent two sequential final Powell minimizations, first in torsion angle space then in Cartesian space. The calculations were repeated multiple times and the lowest energy structure without distance (>0.5 Å) or torsion angle (>5°) violations were used for further refinement steps incorporating a statistical base-base position potential for base-paired nucleotides. The 10-structure energy-minimized structural ensemble was derived from 150 independent calculations using restraints from NOESY experiments and predicted values for A-form helical base-pairs (backbone and ribose dihedral angles, hydrogen-bond and planarity distance restraints), in the presence of the RNA-ff1 force field.

A summary of the NMR restraints used in our structure calculation is provided in Table 1. Convergence was established when we observed no NOE violation >0.5 Å or dihedral angle violations greater than 5° for the majority of structures within the final ensemble. The 3D structures were visualized with PyMol (Schrodinger, Inc.) or Chimera (24) and structural quality was analyzed using Molprobity (25).

**Table 1.** NMR and structural statistics for structure determination of A-paRNA-TL

NMR experimental restraints	
Total number of experimental restraints	2427
Average no. of restraints per nucleotide	25.8
Average no. of NOE-derived restraints per nucleotide	14.3
NOE-derived restraints	1347
Intra-residue	949
Inter-residue	398
Sequential |*i*-*j*| = 1	245
Non-sequential |*i*-*j*| > 1	153
Dihedral restraints	836
Hydrogen-bonding restraints	178
Planarity restraints	66
Structure analysis	
NOE violations(>0.5 Å)	1
Dihedral angle violations (>5°)	0
Bond angle outliers (>5°)	3
Clash score (all atoms)	4.73
Heavy atom RMSD from mean structure, Å	
(including all the internal loops)	
All RNA heavy atoms	2.31
All RNA backbone	2.19

### Small-angle X-ray scattering (SAXS)

RNA samples for SAXS were prepared similarly to samples made for NMR measurements but at the lower concentration range of 1–5 mg/ml. The SAXS data were collected on a SAXS instrument (BioSAXS-2000) at beamline 12-ID-B of the Advanced Photon Source (APS) of Argonne National Laboratory (ANL), SAXS Core facility of Center for Cancer Research (CCR), National Cancer Institute (NCI) of National Institutes of Health (NIH). The particle distance distribution function *P*(*r*) plots were calculated using GNOM (26). Model generation followed a procedure similar to our previous work (16). In summary, low-resolution *ab initio* shape reconstruction was carried out first using DAMMIN (27). A total of 20 models were generated from the GNOM output with DAMMIN using the ATSAS online server (http://www.embl-hamburg.de/biosaxs). The best and final model was selected with a suite of software (DAMSEL, DAMSUP, DAMAVER, DAMFILT and SUPCOMB) (http://www.embl-hamburg.de/biosaxs) for comparison and fitting to the NMR structure (28).

### Site-directed mutagenesis and AGO1-RNA immunoprecipitation

HA-AGO1 expression vector and CDH1 S-paRNA expressing constructs in pcDNA3.1(+) based vectors were described previously (12). The S-paRNA constructs with the A and C allele were mutated to introduce the CC > U change using an In-Fusion HD cloning kit (Takara). Primers for site-directed mutagenesis and PCR amplification are shown in Supplementary Table 2. Plasmids were amplified in DH5α competent cells and purified. Sequences of the wild type and mutated S-paRNA constructs were verified by DNA sequencing. RNA immunoprecipitation for assessing AGO1 binding was performed as previously described (16). PC3 cells were transiently transfected with HA-AGO1 and S-paRNA expression vectors with wild-type and mutated sequences in A and C allelic background. Cells were collected after 48 h and processed using Magna RIP RNA Binding Protein Immunoprecipitation Kit (Millipore). Samples were incubated overnight with Protein A/G Magnetic Beads (Pierce) coupled with antibodies directed to HA-tag (Santa Cruz, F7 SC-7392, 5 μg). Beads were washed and RNA was extracted with an SV Total RNA Isolation System (Promega), with three additional steps of DNase I digestion (Qiagen) to remove any residual plasmid or genomic DNA. Samples were analysed by end-point RT–PCR as described (16). CD44 mRNA, a known AGO1-bound transcript, was used as positive control.

## RESULTS

### Secondary structure of the E-cadherin transcript with the -160A allele

We recorded NMR spectra for the A-paRNA-WT, corresponding to the domain of the sense transcript with the –160A allele. The 1D spectrum contains many NH peaks in the region where base pairs can be monitored (11–14 ppm), revealing a well-folded structure (Figure 1). However, the relatively poor quality of the spectra, as reflected in broad and overlapped cross-peaks in the 2D datasets (Supplementary Figure S10), would prevent structure determination. Below 15°C, increased broadening renders the spectra effectively useless. We have encountered this problem with several other RNAs studied in the laboratory, including the CssA thermometer (16) and the stem loop within the c-JUN 5′ UTR recognized by eIF3 during specialized translation initiation (17). As was done in those projects, we introduced tetraloops in the A-paRNA-WT sequence, in place of three existing loops, to generate A-paRNA-TL, as shown in Figure 1B. These substitutions were based on the SHAPE-generated secondary structure (12), as noted above, and resulted in substantially improved spectral quality (Figure 1C).

To examine whether introduction of the tetraloops altered the RNA structure, we mapped the NH assignments for A-paRNA-TL (with tetraloops), obtained as described below, onto the A-paRNA-WT (wild type loops) spectra. An overlay of the imino region spectra for the two RNAs show many overlapping peaks, indicative of very similar secondary structures for both constructs (Figure 1C). Thus, introducing tetraloops in the A-paRNA-WT sequence does not change its secondary structure but stabilizes it and reduces aggregation, making high-resolution NMR studies of this RNA possible.

**Figure 2.**
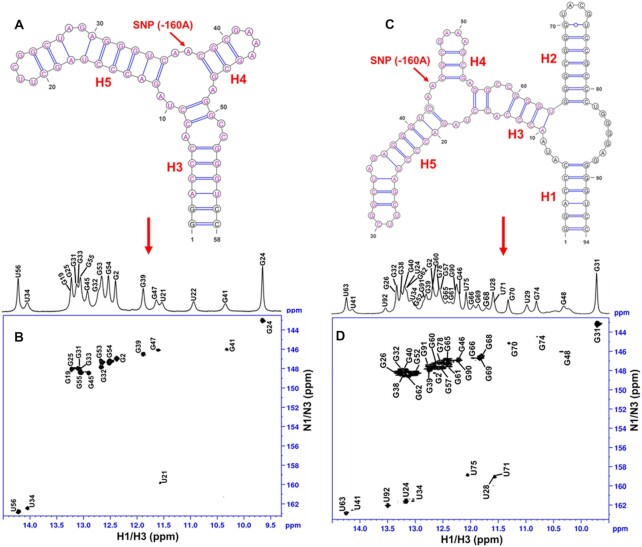
(**A**) Secondary structure of A-paRNA-TL-tr, which was designed to isolate the three-way junction where the SNP is located, as established by NMR; (**B**) 2D ^1^H–^15^N-HSQC spectrum of the same RNA construct recorded at 15°C, with NMR assignments. (**C**) Secondary structure of A-paRNA-TL, as established by NMR; (**D**) 2D ^1^H–^15^N-HSQC spectrum of the same RNA construct recorded at 15°C, with NMR assignments.

The sharp and well-resolved H2 protons allowed us to identify U–A base pairs, while G–C base pairs were identified from the strong cross peaks between GH1 and the pair of Cytidine amino protons. Wobble base pairs were identified by the characteristically strong cross peaks between GNH1 and UNH3 within the non-canonical base-pairing range (12–10 ppm). Based on these attributions, and on the canonical pattern of sequential NOEs involving imino protons in helical regions, we assigned all slowly exchanging NH chemical shifts for the A-paRNA-TL and for a shorter variant lacking the terminal stem-loop (called A-paRNA-TL-tr, Figure 2). The latter construct retains the three-way junction where the SNP is located, as well as the two stem-loops emanating from it (H4 and H5), and helix H3, but the larger three-way junction near the boundary of the domain formed by helices H1, H2 and H3 has been removed.

All assignments were consistent with the predicted secondary structure, and all base pairs predicted from the SHAPE analysis (12) were observed, except for nucleotides that are unpaired or paired but at the ends of helices; overall, the results are fully consistent with the secondary structure generated from our previously reported SHAPE analysis.

In order to investigate whether the C/A polymorphism would alter the secondary structure, we prepared the C-paRNA-WT construct, corresponding to the domain of the sense transcript with the −160C allele in place of -160A (Fig S11). The 1D spectra for the C-paRNA-WT contain numerous NH peaks in the region where base pairs can be directly monitored (11–14 ppm) as well, revealing a well-folded structure (Supplementary Figure S12A). However, due to the relatively poor quality of the spectra, as reflected in Supplementary Figure S12A, we introduced tetraloops in the C-paRNA-WT sequence in place of existing loops, based again on the SHAPE-generated secondary structure (12), to generate C-paRNA-TL (Supplementary Figure S11).

An overlay of the imino ^1^H NMR spectra for the C-paRNA-TL (-160C allele) and C-paRNA-WT (−160C allele) and a comparison of the 1D imino ^1^H NMR spectra of these two RNAs at 15°C, is shown in figures S11 and S12, with assignments shown for the C-paRNA-TL. The spectra are very similar, indicative of conserved secondary structures, and the spectral quality is improved considerably, as expected.

In contrast, comparison of the 1D imino regions for the A-paRNA-TL (−160A allele) and C-paRNA-TL (−160C) (Supplementary Figure S11) shows that the NMR spectra of the A and C variants differ substantially, reflecting changes in the secondary structure arising from the SNP. To confirm this hypothesis, we used NH assignments (Supplementary Figures S11 and S12) obtained from extensive analysis of NOESY and ^1^H–^15^N HSQC imino NMR spectra to determine the secondary structure of C-paRNA-TL (−160C) (Supplementary Figures S11 and S12). Figures S13 and S14 show a comparison of the NMR-derived secondary structures for the A-paRNA-TL and C-paRNA-TL, each based on NH assignments.

Based on this analysis, we conclude that the region of the two RNAs that comprises the miRNA binding site (helix H2 in Figure 1) adopts the same secondary structure regardless of which allele is present at position −160. Furthermore, the longer stem-loop emanating from the three-way junction where the SNP is located (helix H5 in Figure 1) also retains the same secondary structure, as does helix (H1) that defines the boundary of the domain. However, the single A→C change at position -160 alters the structure of the junction, resulting in a new secondary structure for the junction itself, for helix H4 and for H3, the helix that links the SNP to the domain that recruits the miRNA. As a consequence of the rearrangement of helix H3, the other three-way junction is also remodeled, such that helix H2 now emanates from a larger multi-helix junction. This new structure changes the thermodynamic and/or kinetic stability of helix H2, allowing differential access by isomiR-4534/AGO1 complex, which initiates assembly of SUV39H1 (see below).

In summary, analysis of the NMR spectra of paRNA domains containing either allele demonstrates that both RNAs are well-folded, adopting a secondary structure consistent with the SHAPE analysis (12), and that the secondary structures of the transcripts of the two polymorphic sequences differ significantly in the region linking the SNP to the isomiR binding site. To understand which structural features of the RNA are responsible for the conformational differences, we proceeded to determine the three-dimensional structure of the A-paRNA-TL construct.

### ‘Divide-and-conquer’ allows nearly complete spectral assignments and the collection of a large NOE dataset for structure determination

The quality of the NMR spectra of the A-paRNA-TL construct is high, considering the size of the RNA, but it remains very challenging to obtain complete spectral assignments and collect a large number of NOEs, due to fast relaxation and the resulting peak broadening, as well as very crowded NMR spectra. 3D and higher dimensionality experiments based on ^13^C editing are of somewhat limited use due to fast relaxation, as is segmental labeling (29), which would reduce spectral overlap but not the broad lines. Combination of limited experimental NMR data (e.g. a small set of NOEs from slowly relaxing protons like AH2 obtained by extensive perdeuteration and selective carbon labeling) with RDC’s and SAXS can be used to generate 3D structural models, but these approaches rely extensively on modeling assumptions to overcome the paucity of direct experimental observations (30–33). Unsurprisingly, the quality of NMR structures of RNA improves significantly when the density of experimental NOEs is high (34,35).

To address these limitations, we used the ‘divide and conquer’ approach to complete chemical shift assignments and obtain a large number of NMR constraints for structure determination, by starting with smaller independently folded sub-domains. This approach, illustrated in Figure 2 and Supplementary Figure S1, is grounded in the modular nature of RNA structure; RNA secondary structure domains fold independently, while tertiary interactions stabilize but only seldom rearrange the secondary structure (21,22,36). Accordingly, four smaller constructs of varying length were generated (18–58 nts; Supplementary Figure S1). When the overlapping elements of these smaller constructs are superposed, the independent sub-domains can be aligned and combined to generate the complete A-paRNA-TL (Supplementary Figures S1 and S3). Two of the larger fragments (called A-paRNA-TL-tr and A-paRNA-TL-tr-1) contain the 3-way junction formed by helices H3, H4 and H5 (Figures 1 and 2) where the A/C SNP is located and isolate it within smaller RNA constructs to reduce spectral overlap and allow closer investigation of its structure. The stem-loop derived from it (A-paRNA-TL-tr-2) contains the longer stem-loop emerging from the three-way junction, helix H5. Finally, construct A-paRNA-2 isolates the isomiR-4534 binding site, helix H2.

When we examined the isolated sub-domains, we observed all of the imino peaks in the 1D (Supplementary Figure S2) and 2D NMR spectra that would be expected based upon the secondary structures shown in Figures 1, 2 and Supplementary Figure S1; relevant illustrative spectra are shown in Supplementary Figures S15–S18. Superposition of the 1D ^1^H and 2D NOESY NMR spectra (with and without perdeuteration) of the smaller subdomains on those of the full A-paRNA-TL spectra revealed a close correspondence between the chemical shifts and NOESY cross-peaks for both the exchangeable and non-exchangeable protons of each construct (Supplementary Figures S2-S4), demonstrating that the structure found in the complete A-paRNA-TL is retained in each fragment. Once the secondary structure of each fragment was verified, we recorded 2D and 3D-NOESY spectra at different mixing times for both exchangeable and non-exchangeable protons to obtain nearly complete and unambiguous chemical shift assignments. To achieve further simplification of the spectra, we also prepared perdeuterated RNA samples of the three larger fragments, A-paRNA-tr-1, A-paRNA-tr-2 and A-paRNA-tr (representative sections of the spectra are shown in Supplementary Figures S6–S8) and compared the resulting NMR spectra with the spectra of the perdeuterated A-paRNA-TL (Supplementary Figures S4 and S9).

Briefly, imino proton assignments for A-paRNA-TL establish its secondary structure and were initiated with the U28–G32, G70–U75 and U71–G74 wobble base pairs identified by strong cross peaks between GH1 and UH3 within the non-canonical base-pairing range (12–10 ppm) (Supplementary Figure S19). A series of NOESY experiments collected in D_2_O with mixing times between 100 and 300 ms were used to assign the non-exchangeable protons. The overlap in the NMR ribose spectra was resolved through deuteration of the H3′, H4′ and H5′/5″ positions in the sugar and the H5 positions of C/U, which simplified the spectra and sharpened the linewidths by reducing dipolar relaxation (Supplementary Figure S5). Nearly complete spectral assignments were obtained in this manner. Altogether, with the aid of deuteration and 3D ^13^C-edited NOESY spectra, we assigned 91.5% of resonances for A-paRNA-TL (significant exceptions are the H3′, H4′, H5′ and H5″ peaks for residues U8, U24, A25, A35, G36, A55, C58 and U82, which we were unable to assign). Based on these assignments, we then generated a comprehensive list of distance restraints for each of the individual fragments, which were merged to generate a restraint table for the A-paRNA-TL (Table 1). These restraints were used in the structure calculation of A-paRNA-TL.

### Structure analysis and validation

**Figure 3.**
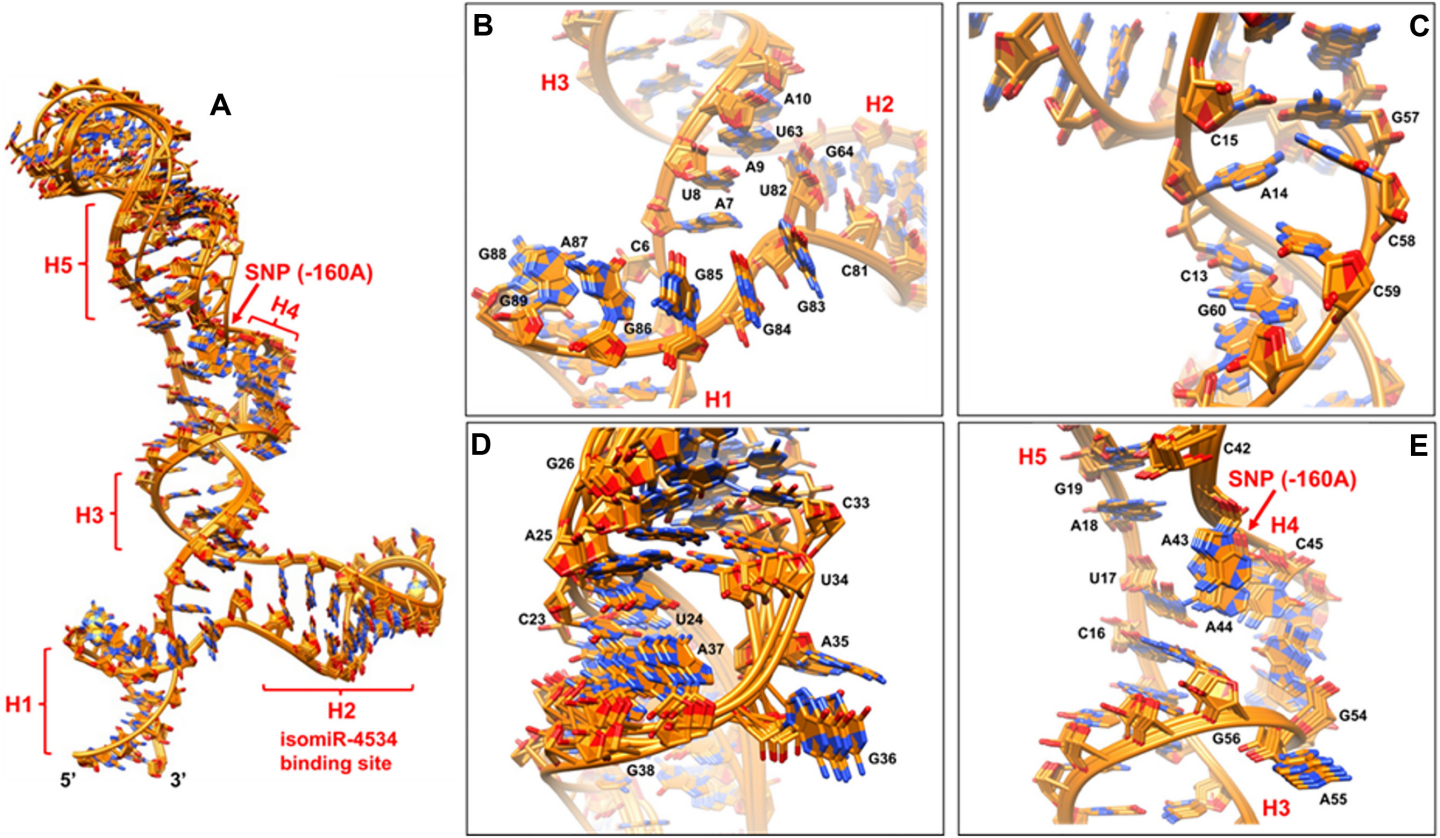
(**A**) Ten lowest-energy structures superposed to generate the structural ensemble of A-paRNA-TL. (**B**) Close up view of the 3-way junction from which the isomiR binding site emanates, formed by helices H1, H2 and H3. The unpaired bases G83-G89 stack on top of each other, while U82 is bulged out and U8 is inserted between residues A7 and A9 but not bulged out; as a result, helices H1 and H2 are coaxially stacked while helix H3 is in an orthogonal orientation; (**C**) Close up view of the 13–15; 57–60 internal loop within helix H3. The unpaired A14 nucleotide is inserted between the G60–C13 and G57–C15 base pairs, and the two bases on the opposite strand, C58 and C59, are displaced from the helix to accommodate imperfect stacking of the G60–C13 and G57–C15 base pairs; (**D**) in the internal loop within helix H5, A35 and G36 are transiently bulged out to accommodate the U34–A25 and U24–A37 base pairs; this region of the structure displays relatively high flexibility, resulting in less precisely defined local structure. (**E**) Close up view of the three-way junction formed by helices H3, H4 and H5, where the SNP is located. A43 and A44 stack on top of each other and A55 is bulged out, while U17 and A18 are sandwiched between the G56–C16 and G19–C42 base pairs; as a result, helices H3 and H5 coaxially stack while the third helix H4 is in an orthogonal orientation.

The ‘divide and conquer’ approach allowed us to obtain a much larger number of constraints for structure determination than would have been possible if we had studied the complete RNA. These physical limitations cannot be overcome by segmental or residue-selective isotopic labeling (29). We generated the restraint set (Table 1) by combining NOE-derived distance and torsion angle restraints from each fragment and the structure was then calculated using a well-tested simulated annealing protocol (as discussed in methods section) within NIH-XPLOR. A final ensemble of the 10 lowest energy structures (Figure 3A) from a refined set of 150 structures was chosen for analysis. The overall RMSDs (including all internal loops and three-way junctions) for backbone and heavy atoms were 2.19 and 2.31 Å, respectively. We attribute this relatively low RMSD value to the high number of NOE restraints per nucleotide that we were able to obtain by building the final restraint sets from sets collected for individual subdomains that overlap to generate the full RNA. The individual helical sections of the RNA all show RMSDs below 1.00 Å; for example, helix H1 has an RMSD = 0.95 Å and helix H2 converges to an RMSD of 0.90 Å (Figure 3A). An exception is presented by the flexible tip of helix H5 (Figs. 2C and 3D), near the internal loop formed by nucleotides 24–25/34–37, whose bases are transiently in a solvent exposed conformation that affects the neighboring base pairs as well. This region is less well constrained (Figure 3D) due to the smaller number of available NOEs and shows a higher local RMSD value of 2.95 Å. Likewise, bases located within the SNP three-way junction formed by helices H3, H4 and H5 (nucleotides 16–19, 42–45 and 54–56), also converged less well (Figure 2C and Figure 3E) due to the paucity of NOEs between loop residues, leading to a locally higher RMSD of 1.98 Å. In contrast, the internal loop in helix H3 (involving nucleotides 13–15 and 57–60), which connects the two three-way junctions (Figures 2C and 3C) shows a low RMSD of 1.19 Å, yet it remains conformationally dynamic, as evidenced by mixed sugar conformations, as discussed in the next paragraph. Likewise, the purine-rich three-way junction linking helices H1, H2 and H3 and involving nucleotides 6–10, 63–64 and 81–89, from which the isomiR-binding stem–loop H2 emanates (closed by the U63–A10, G64–C81 and G89–C6 base pairs, Figures 2C and 3B) superposed well (purines G83, G84, G85, G86, A87, G88 and G89 all stack on top to each other) and shows a low local RMSD of 0.92 Å.

For further validation of the NMR-derived 3D structure of A-paRNA-TL, we collected SAXS data. The NMR-generated lowest energy structure of A-paRNA-TL fits well within the SAXS model (see Supplementary Figure S20A) supporting the overall shape and arrangement of the NMR-derived A-paRNA-TL structure. We also validated the NMR structure of the shorter A-paRNA-TL-tr variant, which lacks the terminal stem-loop (Figure 2A). The NMR-generated lowest energy structure of A-paRNA-TL-tr also fits well within the SAXS model (see Supplementary Figure S20B); the SAXS results were not used for NMR refinement, and therefore the good agreement between the results of the two methods provides independent validation of the NMR structure.

We assessed the presence of conformational dynamics only by analyzing the sugar conformation, because it would be challenging to conduct ^13^C relaxation studies on such a large RNA. Nearly all nucleotides within the A-paRNA-TL construct show 3′-endo sugar pucker conformation (we ignore in this discussion residues within the tetraloops whose properties have been described exhaustively in the past). Exceptions include A14 (within the 13–15 and 57–60 internal loop in helix H3); A35 and G36 (in the 24–25/34–37 internal loop within helix H5); A55 (within the SNP three-way junction), as well as U82 within the other three-way junction. All of these residues exhibited strong H1′–H2′ and H1′–H3′ TOCSY correlations, suggesting a 2′-endo sugar pucker conformation. In addition, C58 and C59 (within the 13–15 and 57–60 internal loop in helix H3, Figures 2C and 3C) and U17 (part of the SNP three-way junction, Figures 2C and 3E) exhibit mixed 2′-endo/3′-endo sugar pucker conformations, based on relatively strong H1′ to H2′, H3′ and H4′ cross-peaks. They also display deviations from A-form helical pattern in the phosphodiester backbone, consistent with the presence of local conformational dynamics. This is reflected in higher local uncertainty in the structure and higher RMSD, as discussed in the previous paragraph. As is discussed next, the conformational flexibility of both the three-way junction where the SNP is located, as well as the internal loop in helix H3, are likely to be functionally significant.

### 3D structure of the CDH1 paRNA (−160A SNP)

Examining the structure starting from its 5′- and 3′-ends that come together to form helix H1 that defines the boundaries of the domain, a first prominent feature is the purine-rich three-way junction abutted by helices H1, H2 and H3 (Figures 2C and 3B). No evidence of stable cross-strand base pairing was found based on the absence of NOEs between guanine NH_2_ or AH2 to the cross strand H1′, and the observation of no slow exchanging NHs. However, the stretch of purine bases (G83-G89) all stack on top to each other (Figure 3B), as reflected in the characteristic sequential pattern of NOEs from H1′/ H2′ to H6/H8 (Supplementary Figure S21) observed in the highly deuterated sample; only residue U82 is bulged out and adopts the 2′-endo sugar conformation that extends the phosphodiester backbone. U8 is inserted between A7 and A9 but not bulged out, allowing for stacking interactions between the two adenines (Figure 3B). As a result, helices H1 and H3 stack coaxially, while the third helix (H2) is oriented approximately orthogonally to the coaxial stack (Figure 3B). This topological arrangement matches the ‘family A’ topology (37), a very common tertiary motif in three-way RNA junctions (37,38).

The most important functional feature of the A-paRNA-TL is the three-way junction formed by helices H3, H4 and H5 (Figure 2C and Figure 3E), where the SNP is located. Within this junction, residues A43 and A44 (the latter of which coincides with the SNP-160A) stack on top of each other while A55 is bulged out and adopts the 2′-endo sugar conformation (Figure 3E); residues U17 and A18 are sandwiched between the G56–C16 and G19–C42 base pairs (Figure 3E). In the NMR structure, residue A44 is unpaired and stacked on A43 but does not form any cross strand interactions. Pairs of unpaired As such as this are often found in 3-way junctions and internal loops, for example in ribosomal RNAs (39,40). These residues often orient and stabilize coaxially stacked helices at three-way junctions and are important for the formation of tertiary motifs in these RNAs (39,40).

This ‘SNP-three-way’ junction shows a topology and arrangement of helices similar to the first three-way junction, with helices H3 and H5 coaxially stacked and the third helix (H4) approximately orthogonal to them (Fig 3E). However, this second three-way junction (Fig 3E) is structurally less rigid than the first (Figure 3B, E), with a higher local RMSD of 1.98 Å, caused in part by the conformational dynamics observed for U17, which experiences a mixed sugar conformation. In the C-paRNA-TL (−160C), the AA motif is changed to AC, and the entire three-way junction is remodeled, in conjunction with helix H3 linking it to the larger three-way junction near the isomiR binding site. As a result, a larger four-way junction forms (Supplementary Figure S11).

This reorganization is most likely facilitated by the small internal loop within H3 just two base pairs away from the SNP junction. This loop introduces a structural distortion that destabilizes helix H3, and is seen to be dynamic, as revealed by the mixed sugar conformation observed for residues C58 and C59 (numbers refer to the A-allele). In this internal loop, A14 is inserted between the G60−C13 and G57−C15 base pairs (Figure 3C) and the only deviation from A-form helical pattern is found in the C15 backbone angles, as required to accommodate A14. The two bases opposite, C58 and C59, however, are displaced from helical stacking and experience conformational exchange between 2′-endo and 3′-endo pucker. The three unpaired bases A14, and especially C58 and C59, provide conformational flexibility to the internal loop and destabilize helix H3, which could facilitate the rearrangement of the entire helix when the A–C mutation occurs, which is necessary to remodel the secondary structure at the three-way junction when A is changed to C. These results suggest that the SNP three-way junction and the structural destabilization created by the internal loop several base pairs away, are both required for the conformational change to occur when −160A is changed to −160C; this hypothesis is tested below.

Finally, the fourth significant feature in the 3D structure is a small internal loop in helix H5 (five nts) involving nucleotides 24–25 and 34–37 (Figures 2C and 3D). This is the most flexible region of the RNA. Nucleotides A35 and G36 are transiently solvent exposed, leading to relatively poor convergence in the structure for the neighboring base pairs as well (Figure 3D). However, for both allelic variants the entire helix retains the same structure, suggesting that it is not at all likely to be important for differential regulation of the promoter activity.

### The stability of helix H3 affects the conformation of the paRNA and its function in regulating E-cadherin expression

To test the hypothesis that the stability of the helix H3 internal loop affects the organization of the S-paRNA domains, we synthesized two additional RNAs with wild type loops corresponding to either A- or C-containing paRNA, truncated to helix H3 (Supplementary Figure S22). C58 and C59 are replaced with U in each of the two RNAs (CC → U) to create perfectly base paired helices which contain either the A- or the C-allele at the neighboring three-way junction. The 1D ^1^H NMR (Supplementary Figure S23) and 2D ^1^H–^1^H NOESY imino NMR spectra (Supplementary Figures S24–S26) of the three RNAs are very similar, except for the appearance of a strong U NH signal, corresponding to the new AU base pair created by the mutation in helix H3. Thus, once helix H3 is stabilized by replacing unpaired nucleotides within the internal loop to create a perfect helix, the A–C substitution at position −160 has no effect on the secondary structure.

An implication of this result is that a variant of helix H3 containing a stabilized fully helical structure unable to undergo the conformational rearrangement would behave functionally similarly to the A-allele, regardless of whether A- or C-alleles are present. To test the functional implications of the helix H3 stability changes, we used S-paRNA expressing constructs and RNA immunoprecipitation (RIP) to probe the ability of wild type and mutated transcripts to bind AGO1. Vectors with wild-type or mutated (CC → U) A- and C-S-paRNA were co-transfected with the HA-tagged AGO1-expressing vector in PC3 cells. RIP was performed using an anti-HA antibody to specifically pull down AGO1 in co-transfected cells (12). As expected, the A- and C- wild type S-paRNAs showed clearly different binding to AGO1, with minimal affinity for the C transcript (Figure 5). Notably, as predicted by the structural analysis, both the A- and C-paRNAs with the CC → U mutation behaved similarly to the wild-type A-paRNA and bound AGO1 similarly, meaning that altering the stability of the internal loop reduces the difference between -160 A and −160 C at both the structural and the functional level (Figure 5).

## DISCUSSION

E-cadherin acts as a tumor suppressor in many epithelial carcinomas. Its reduced expression is a hallmark of the epithelial-to-mesenchymal transition, which promotes stemness, invasion and metastasis and negatively impacts responsiveness to treatment (6–8). A mechanism of E-cadherin gene silencing involves the recruitment of histone-modifying enzymes to the CDH1 promoter inducing a repressive chromatin state (41). This process is regulated by a S-paRNA that is transcribed from an upstream transcription start site independently of the main promoter (12)). As illustrated schematically in Figure 1A, silencing of the E-cadherin gene requires formation of a miRNA-guided AGO1 complex on the S-paRNA. This complex then recruits the SUV39H1 methyltransferase that induces repressive chromatin modifications, thereby reducing E-cadherin expression. Within this non-coding RNA, an A/C SNP at position −160 from the CDH1 transcription start site is associated with significantly increased prostate cancer risk as a result of increased recruitment of epigenetic enzymes and reduced activity of the CDH1 promoter in the presence of the A allele (12). However, the SNP is distant from the miRNA-AGO1 binding site in primary sequence and secondary structure, raising the question of how this single nucleotide change can affect transcriptional regulation. A possibility is that the effect of the SNP occurs at the structural level, but the role of RNA structure in non-coding RNA function has been suggested much more often than it has been demonstrated, with some notable exceptions (42,43).

**Figure 4.**
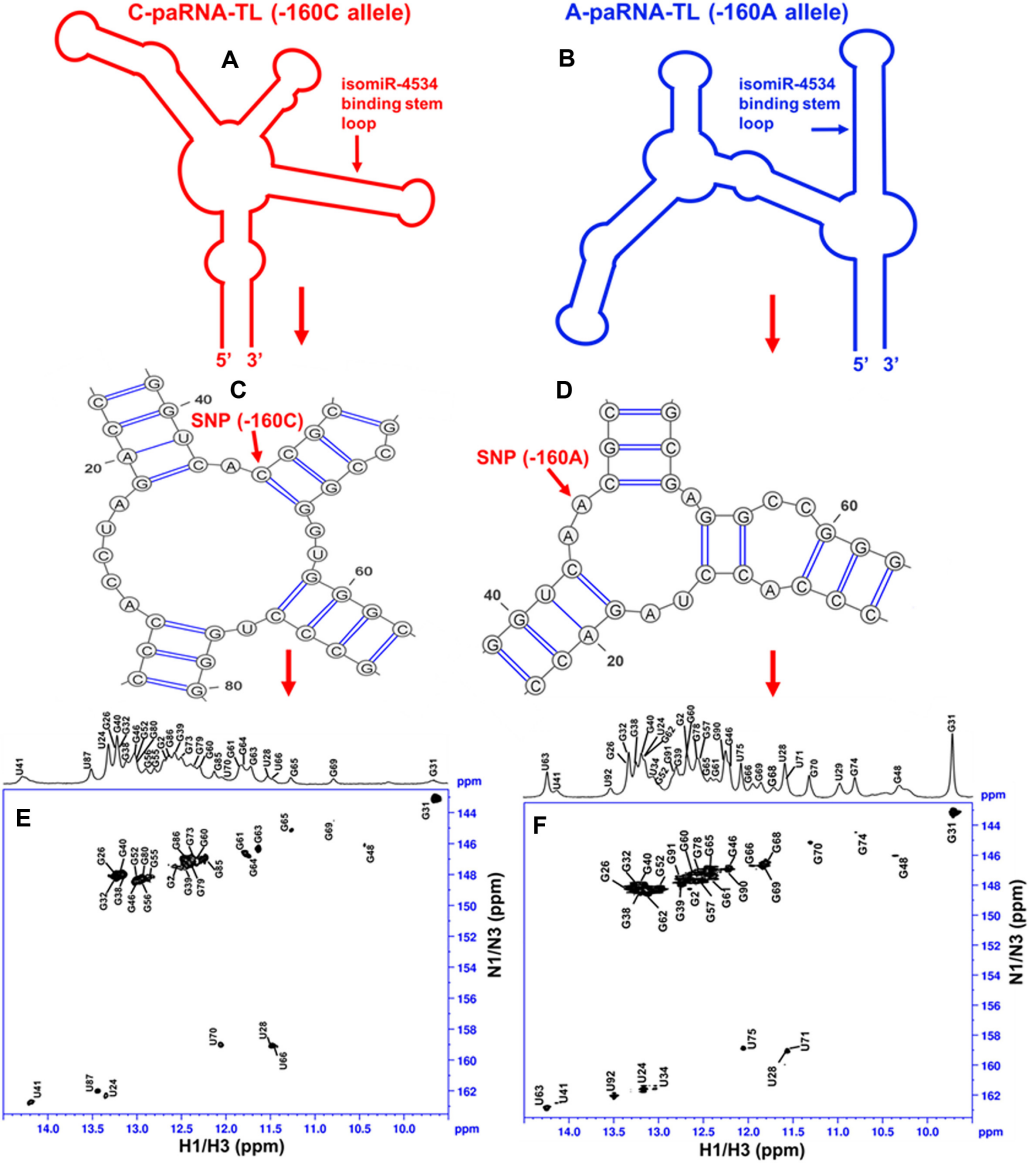
Schematic diagram showing the impact of SNP rs16260 on the structure of the CDH1 paRNA. (**A**) and (**B**) are cartoon representations of the secondary structures derived by NMR and SHAPE for the C- and A-paRNAs. (**C**) and (**D**) are close up views of the of the local secondary structure rearrangement induced by the C to A mutation at position −160. (**E**) and (**F**) are 2D ^1^H–^15^N-HSQC spectra of C-paRNA-TL and A-paRNA-TL recorded at 15 °C, with NMR assignments.

**Figure 5.**
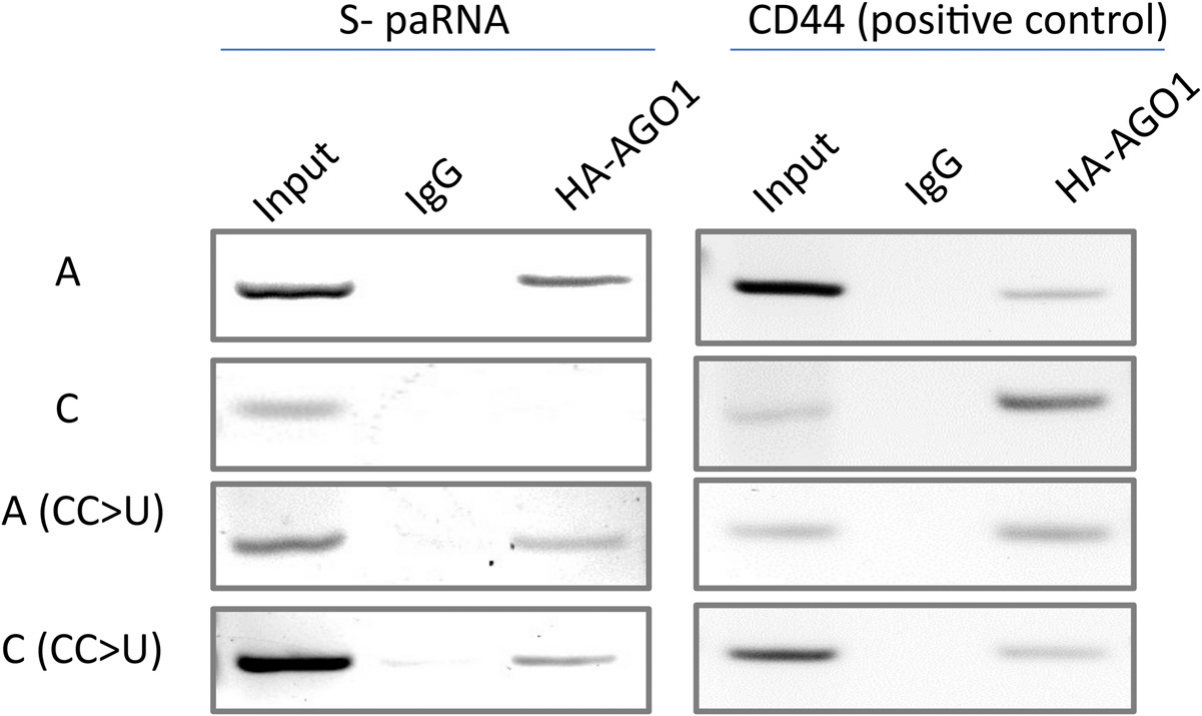
Binding of AGO1 to the S-paRNA with the A or C allele, with or without the CC → U substitution. Samples from PC3 cells were co-transfected with the S-paRNA and HA-AGO1 expression vectors, immuno-precipitated using an anti-HA antibody, and analyzed by RT-PCR. CD44 was used as a positive control for AGO1-bound mRNA.

In order to address the structural role of the SNP in the regulation of E-cadherin, we determined the three-dimensional NMR structure of the 90-nucleotide domain within the paRNA surrounding the SNP and adjacent to the miRNA-AGO1 binding site. This was done using a divide and conquer approach which allowed collection of a large number of experimental constraints (Supplementary Figure S27) and high structural precision. We show here that the A-paRNA-TL folds into a well-defined three-dimensional structure, and that the structures of A- and C-variant SNPs differ significantly. This conformational change extends from the three-way junction where the SNP is located, to promote reorganization of the adjacent helix H3, propagating the effect of the single nucleotide change to the larger three-way junction from which the isomiR-4534 binding site emanates (12). The conformational rearrangement is enabled by the disruption of the structure of the three-way junction as result of the A- to C- change (Figure 4). Notably, the presence of a second structural distortion nearby, the asymmetric internal loop in helix H3 that is only two base pairs away from the junction, facilitates the reorganization of the entire helix. This conformational change is facilitated by the intrinsic instability of this internal loop, compared to a fully paired helix, and by the local conformational flexibility observed for both the three-way junction and the internal loop (Figure 4). In fact, when the helix is stabilized by mutating the internal loop into a perfectly paired helix, the NMR spectra of constructs bearing the A- and C-alleles are very similar (Supplementary Figures S23-S26), implying very similar structures (Supplementary Figure S22), and the C-paRNA behaves like the A-paRNA in the presence of a perfectly paired helix H3 (Figure 5). Thus, the internal loop facilitates reorganization of the three-way junction and helix H3 and allows differential epigenetic regulation by the two variant sequences.

Through this structural mechanism, the conformational rearrangement of helix H3 communicates the single nucleotide change to the entire paRNA domain, although the SNP is located approximately 50 nucleotides away from the isomiR-4534 binding site, generating a plausible way to affect recruitment of a miRNA-protein complex that modulates E-cadherin expression. Our NMR structural studies provide the basis for the effect of a single nucleotide change located away from the functional site and demonstrate that even minimal changes in sequence can significantly affect the function of non-coding RNAs by modulating RNA structure (Figures 4 and 5).

These results highlight a new mechanism by which polymorphic sites and somatic mutations in non-coding genomic regions can affect the epigenetic landscape, inviting consideration of therapeutic targeting with small molecules. Our NMR structural studies represent a first step towards the identification of small molecules that could restore E-cadherin expression in epithelial cancers by regulating protein expression at the RNA level.

## DATA AVAILABILITY

NMR assignments of A-paRNA-TL were submitted to BMRB (http://www.bmrb.wisc.edu) under submission ID 51127. The structure of A-paRNA-TL was deposited with the Protein Data Bank (https://www.rcsb.org/) with accession number 7SHX.

## Supplementary Material

gkac875_Supplemental_File
