# Supplementary material for: A functional SNP regulates E-cadherin expression by dynamically remodeling the 3D structure of a promoter-associated non-coding RNA transcript
Source: Nucleic Acids Res. 2022 Oct 16;50(19):11331–43. doi: 10.1093/nar/gkac875 (PMC9638923; doi:10.1093/nar/gkac875)
Supplement: gkac875_Supplemental_File [file gkac875_supplemental_file.docx]

**Supplementary Material**

**A functional SNP regulates E-cadherin expression by dynamically remodeling the 3D structure of a promoter-associated non-coding RNA transcript**

Shrikant Sharma^1^, Giuseppina Pisignano^2^, Jessica Merulla^3^, Carlo V. Catapano^3^ and Gabriele Varani^1,*^

^1^Department of Chemistry, University of Washington, Seattle, WA, USA, 98195-1700

^2^Department of Biology and Biochemistry, University of Bath, Bath, BA2 7AY, UK

^3^Institute of Oncology Research (IOR), Università della Svizzera italiana (USI), 6500 Bellinzona, Switzerland.

e.mail: [varani@uw.edu](mailto:varani@uw.edu)

**Supporting Tables:**

**Table S1.** Sequences of -160 A/C paRNAs used for NMR structural studies; the A/C and CC sites where mutations were introduced are in red and loops are highlighted in blue to emphasize tetraloop substitutions.

| **-160 A/C paRNAs ID** | **RNA sequence** |
| --- | --- |
| A-paRNA-TL (94 nt) | **5’**GGACCCAUAACCCACCUAGACCCUAGCUUCGGCUAGAGGGUCAACGCGAAAGCGAGGCCGGGUGGGCGGGUACGUCCGCCCUGGGGAGGGGUCC**3’** |
| A-paRNA-WT (104 nt) | **5’**GGACCCAUAACCCACCUAGACCCUAGCAACUCCAGGCUAGAGGGUCAACGCGUCUAUGCGAGGCCGGGUGGGCGGGCCGUCAGCUCCGCCCUGGGGAGGGGUCC**3’** |
| A-paRNA-TL-tr (58 nt) | **5’**GGACCCACCUAGACCCUAGCUUCGGCUAGAGGGUCAACGCGAAAGCGAGGCCGGGUCC**3’** |
| A-paRNA-WT-tr (64 nt) | **5’**GGACCCACCUAGACCCUAGCAACUCCAGGCUAGAGGGUCAACGCGUCUAUGCGAGGCCGGGUCC3’ |
| A-paRNA-WT-tr (63 nt)  (CC motif replaced with U) | **5’**GGACCCACCUAGACCCUAGCAACUCCAGGCUAGAGGGUCAACGCGUCUAUGCGAGGUGGGUCC**3’** |
| C-paRNA-WT-tr (63 nt)  (CC motif replaced with U) | **5’**GGACCCACCUAGACCCUAGCAACUCCAGGCUAGAGGGUCACCGCGUCUAUGCGAGGUGGGUCC**3’** |
| A-paRNA-TL-tr-1 (44 nt) | **5’**GGACCCACCUAGACUUCGGUCAACGCGAAAGCGAGGCCGGGUCC**3’** |
| A-paRNA-TL-tr-2 (26 nt) | **5’**GGACCCUAGCUUCGGCUAGAGGGUCC**3’** |
| A-paRNA-2 (18 nt) | **5’**GGGCGGGUACGUCCGCCC**3’** |
| C-paRNA-TL (89 nt) | **5’**GGACCCAUAACCCACCUAGACCCUAGCUUCGGCUAGAGGGUCACCGCGAAAGCCGGGUGGGCGGGUACGUCCGCCCUGGGGAGGGGUCC**3’** |
| C-paRNA-WT (104 nt) | **5’**GGACCCAUAACCCACCUAGACCCUAGCAACUCCAGGCUAGAGGGUCACCGCGUCUAUGCGAGGCCGGGUGGGCGGGCCGUCAGCUCCGCCCUGGGGAGGGGUCC**3’** |

**Table S2:** Sequences of primers used for site directed mutagenesis.

**Supporting Figures:**


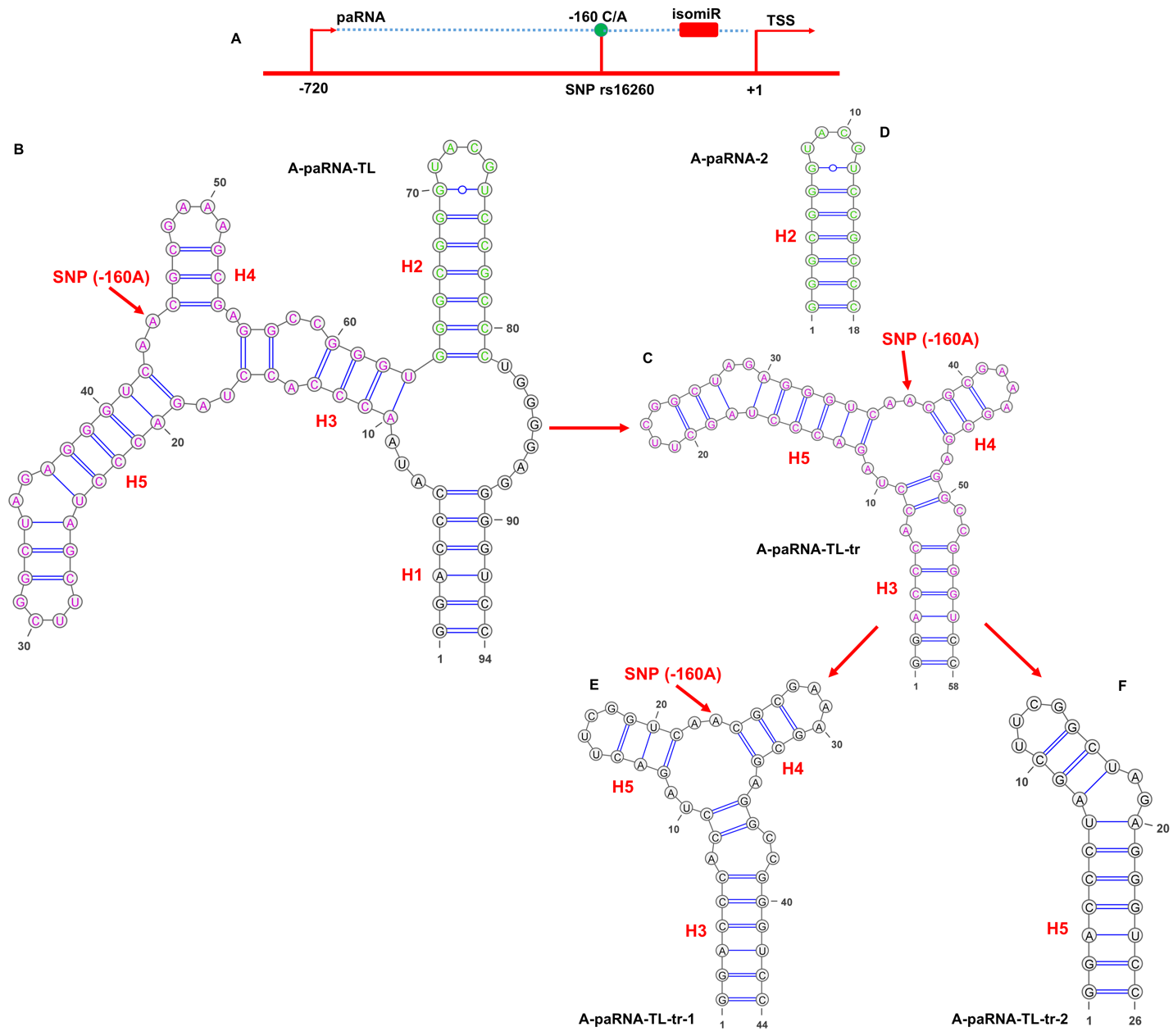


**Figure S1**. A) Schematic diagram of the genomic region that immediately precedes the transcription start site of the CDH1 gene. The independent transcription start site for the paRNA, the SNP rs16260 (green circle), and the isomiR-4534 binding site (red rectangle) are marked; B) SHAPE-derived secondary structure (confirmed by NMR in this study) of the A-paRNA-TL construct, which was divided into four segments (C-F) which overlap to generate the complete structure; these five constructs were all used in the NMR studies.


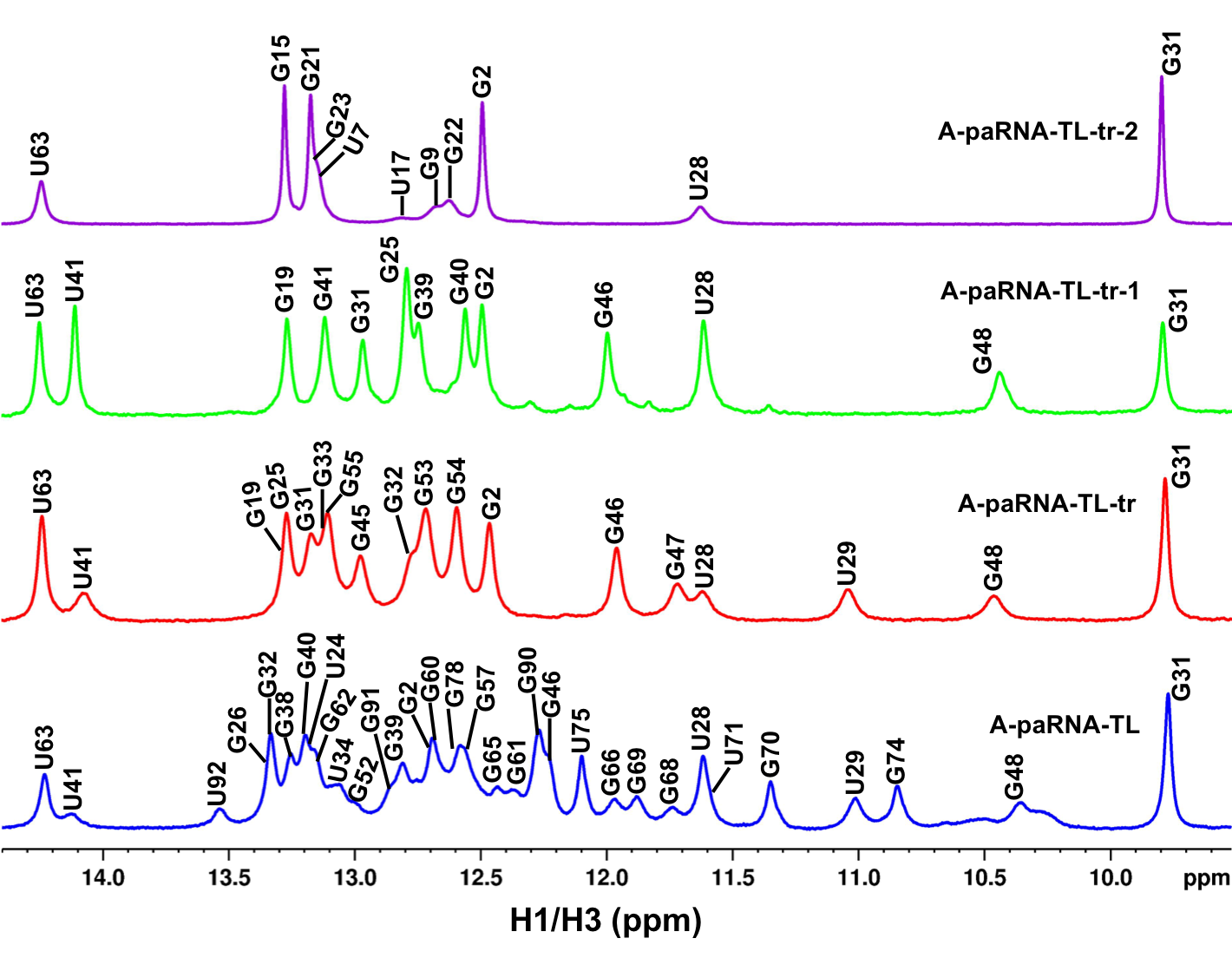


**Figure S2**. Overlay of the 1D ^1^H NMR spectra (imino region only) for the A-paRNA-TL, A-paRNA-TL-tr, A-paRNA-TL-tr-1 and A-paRNA-TL-tr-2 constructs recorded at 15 °C, with assignments. The corresponding secondary structures are shown in Fig. S1. The remarkable similarities in the imino resonances demonstrate the presence of very similar secondary structures across all four RNAs. In other words, the secondary structure of the full A-paRNA-TL is fully preserved in each of the individual fragments.


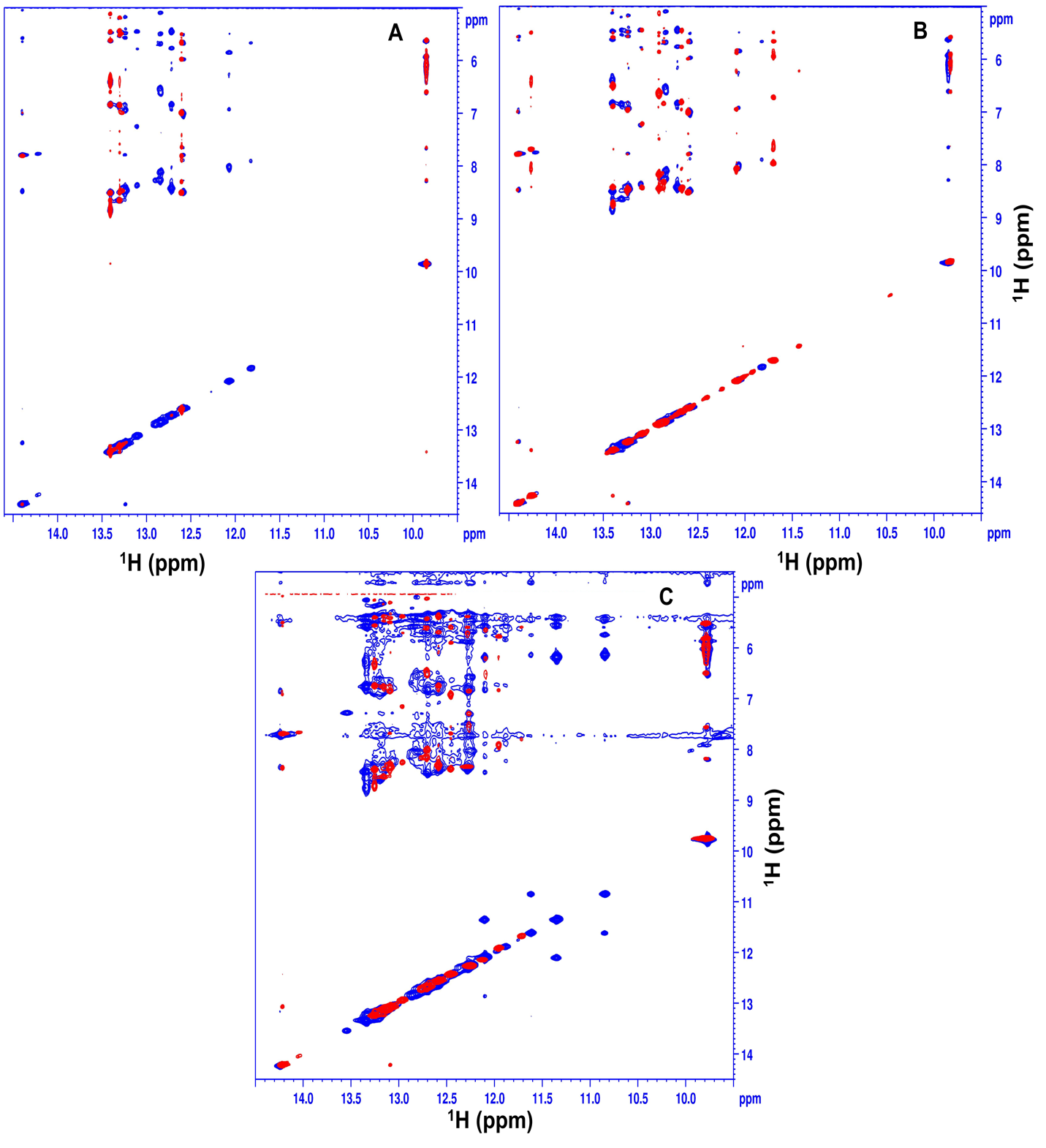


**Figure S3.** Overlays of the imino regions of 2D ^1^H-^1^H NOESY NMR spectra recorded at 15 °C (figure 1 shows the corresponding secondary structures). A) Overlay of A-paRNA-TL-tr (blue) and A-paRNA-TL-tr-2 (red). B) Overlay of A-paRNA-TL-tr (blue) and A-paRNA-TL-tr-1 (red). C) Overlay of A-paRNA-TL (blue) and A-paRNA-TL-tr (red). The considerable similarities in the imino resonance peak positions (as in Figure S2) and in the patterns of NOE cross peaks demonstrate the presence of very similar secondary structures.


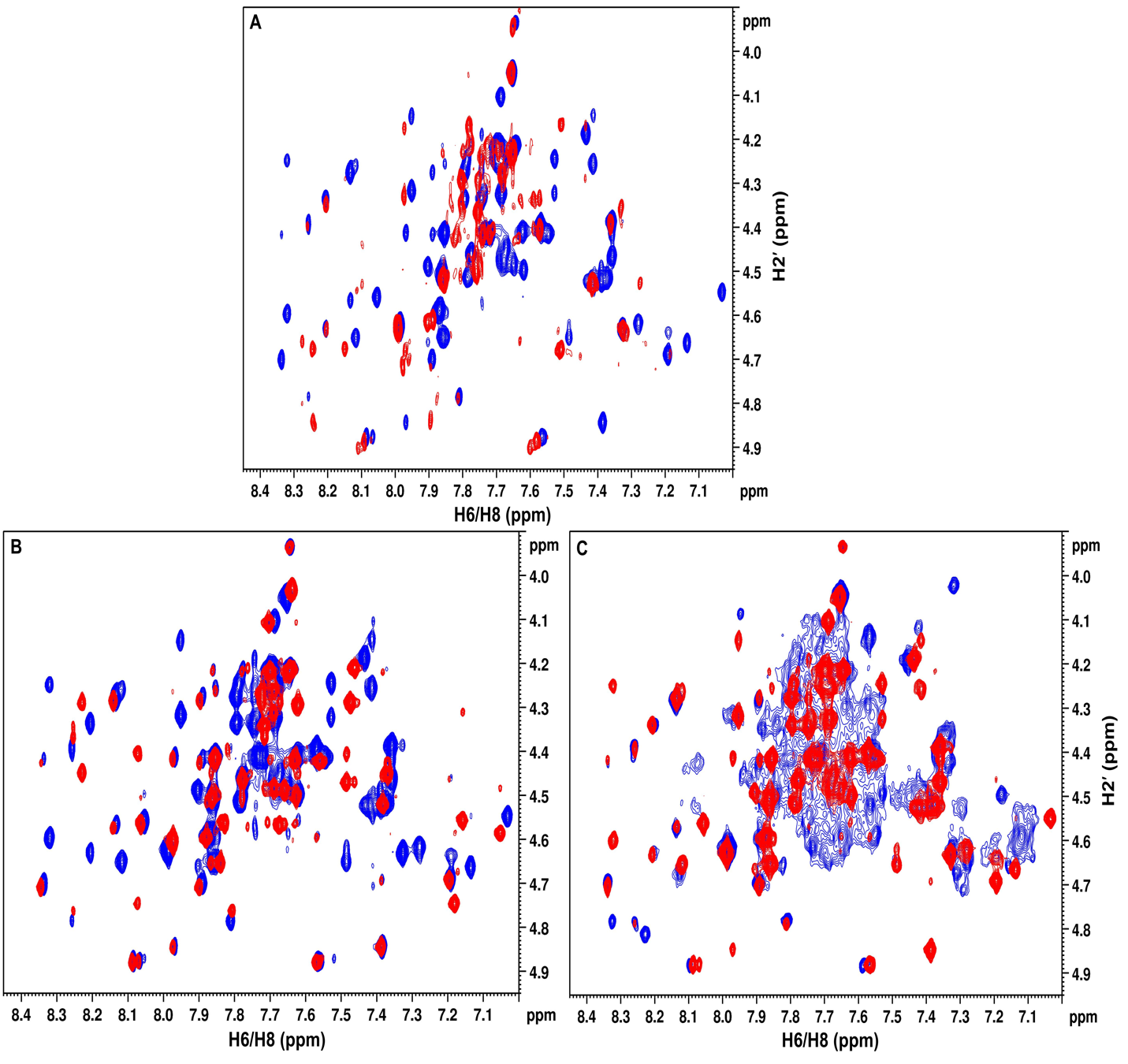


**Figure S4**. Overlay of the D_2_O 2D ^1^H-^1^H NOESY spectra of perdeuterated RNAs recorded at 25 °C, revealing a highly transferable pattern of chemical shifts and NOESY cross peaks. A) Overlay of the H2′ to H6/H8 region of A-paRNA-TL-tr (blue) and A-paRNA-TL-tr-2 (red). B) Overlay of the H2′ to H6/H8 region of A-paRNA-TL-tr (blue) and A-paRNA-TL-tr-1 (red). C) Overlay of H2′ to H6/H8 region of A-paRNA-TL (blue) and A-paRNA-TL-tr (red). Even with extensive deuteration, overlap is considerable in this last spectrum, demonstrating the necessity of the divide-and-conquer approach.

**
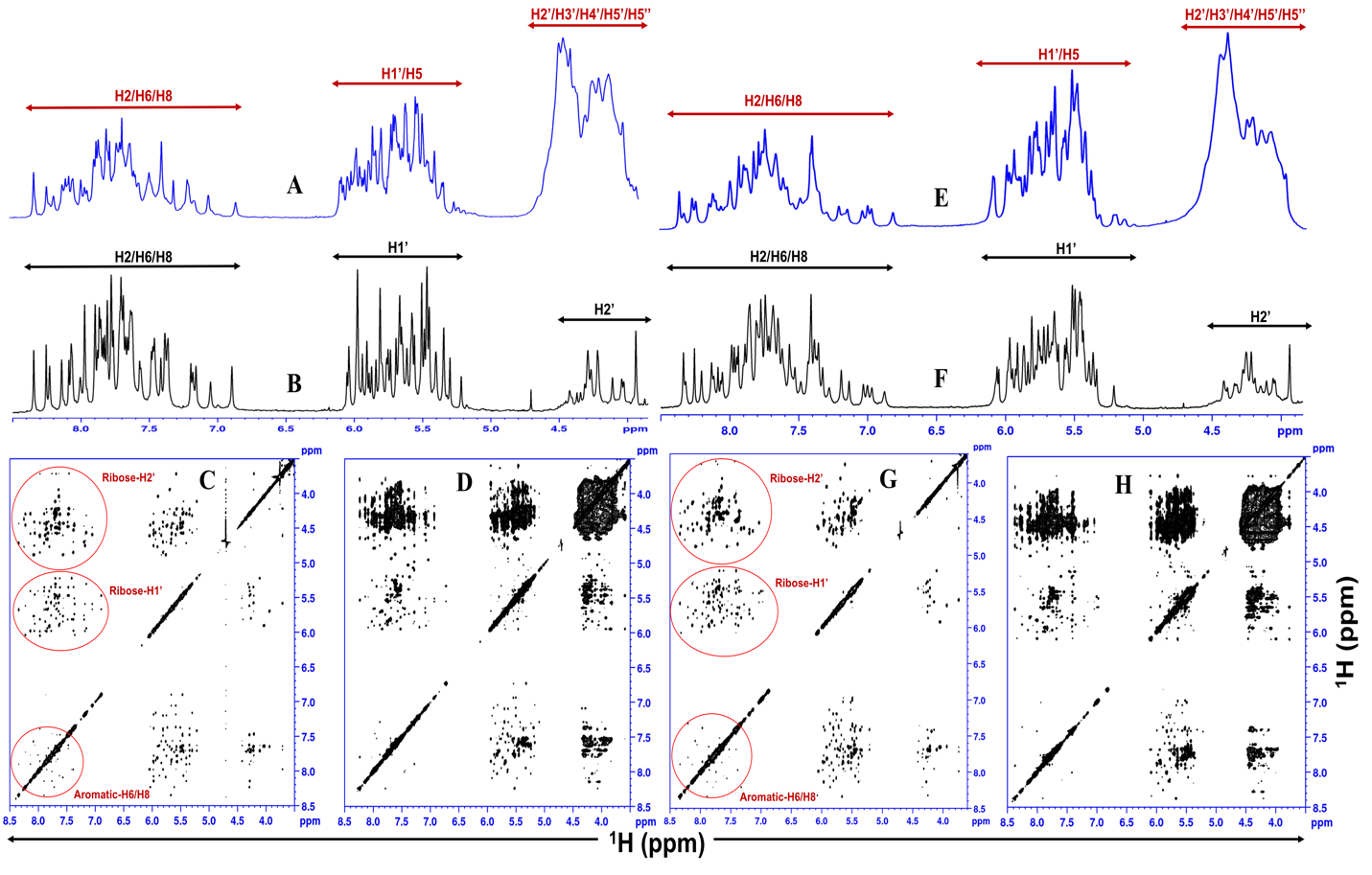
**

**Figure S5.** Base and ribose deuteration greatly improve the quality of the NMR spectra of these relatively large RNAs. 1D ^1^H NMR spectra collected at 25 ^°^C without A) and with B) (H6/H8, H1′, H2′, D3′, D4′, D5′/D5′′and D5) ribose deuteration. C) and D) are 2D ^1^H-^1^H NOESY spectra recorded at 25 °C for the A-paRNA-TL-tr-1. E) and F) show 1D ^1^H NMR spectra with and without (H6/H8, H1′, H2′, D3′, D4′, D5′/D5′′ and D5) ribose deuteration). G) and H) show 2D ^1^H-^1^H NOESY spectra with G) and without H) selective deuteration for the A-paRNA-TL-tr.


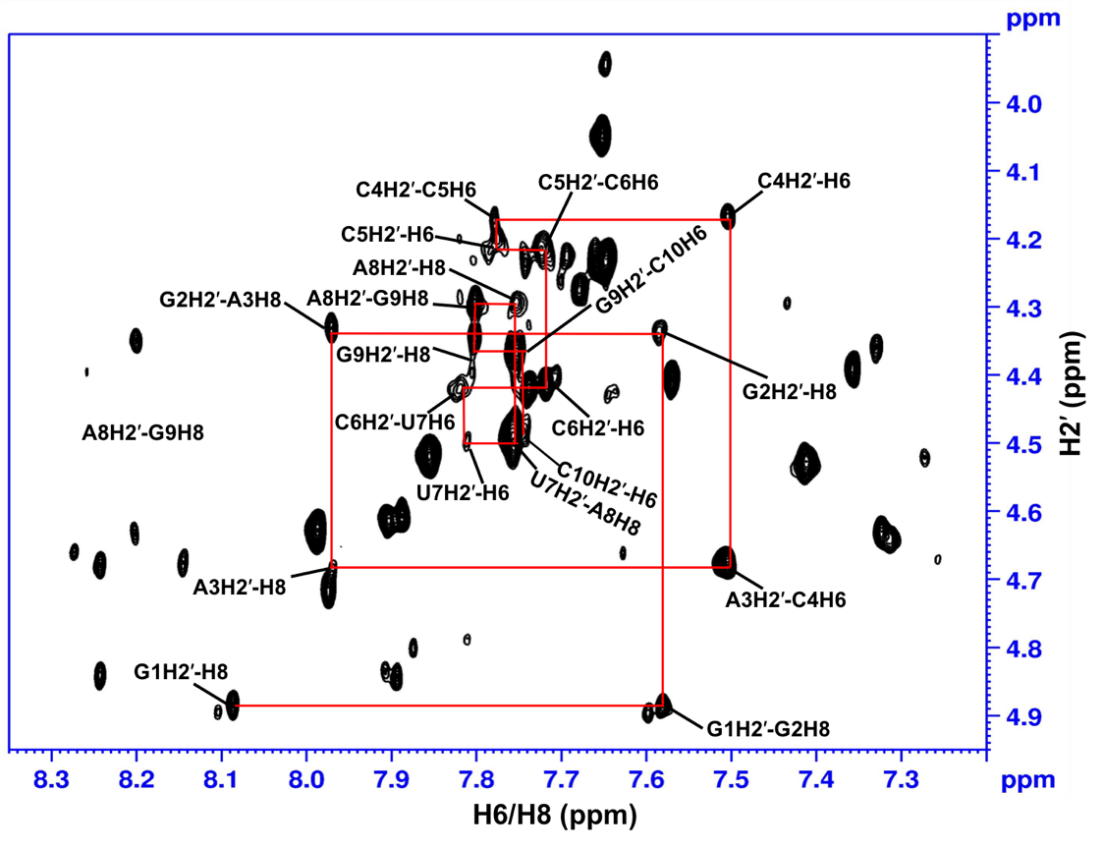


**Figure S6**. The H2′ to H6/H8 ‘helical walk’, annotated from G1 to C10, for A-paRNA-TL-tr-2, plotted on a 2D ^1^H-^1^H NOESY spectrum recorded at 25 °C with (H6/H8, H1′, H2′ but D3′, D4′, D5′/D5′′ and D5) ribose deuteration.


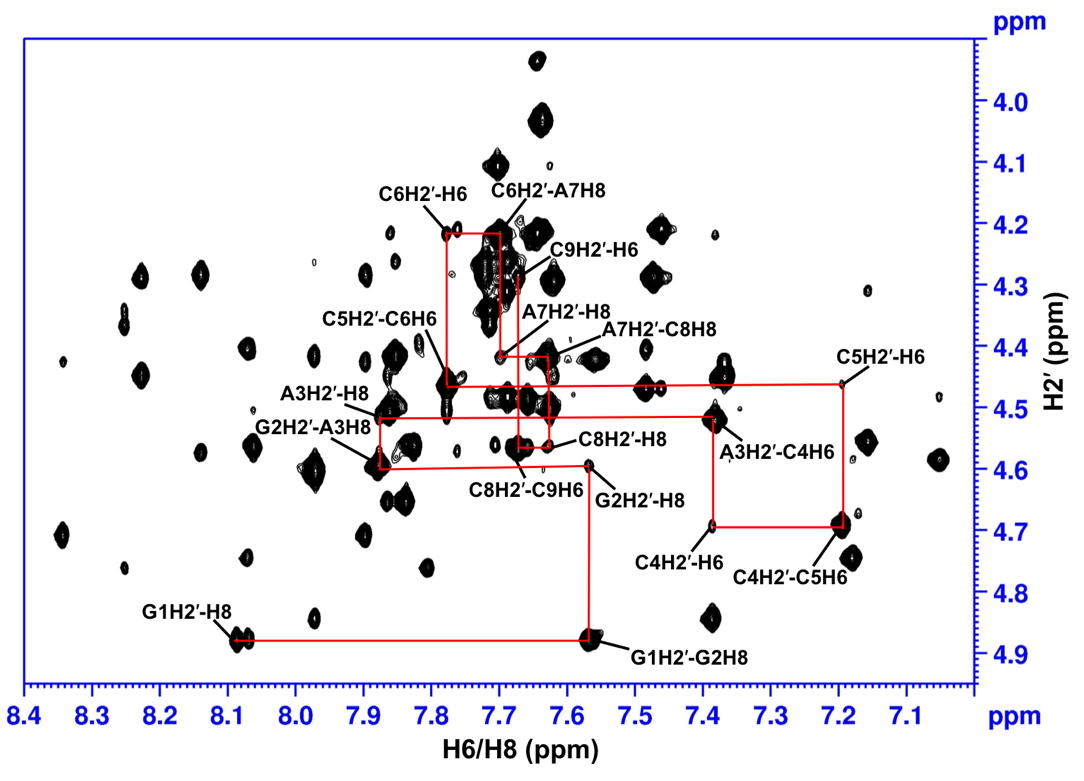


**Figure S7.** The H2′ to H6/H8 ‘helical walk’, annotated from G1 to C9, for A-paRNA-TL-tr-1, plotted on a 2D ^1^H-^1^H NOESY spectrum recorded at 25 °C with (H6/H8, H1′, H2′ but D3′, D4′, D5′/D5′′ and D5) ribose deuteration.


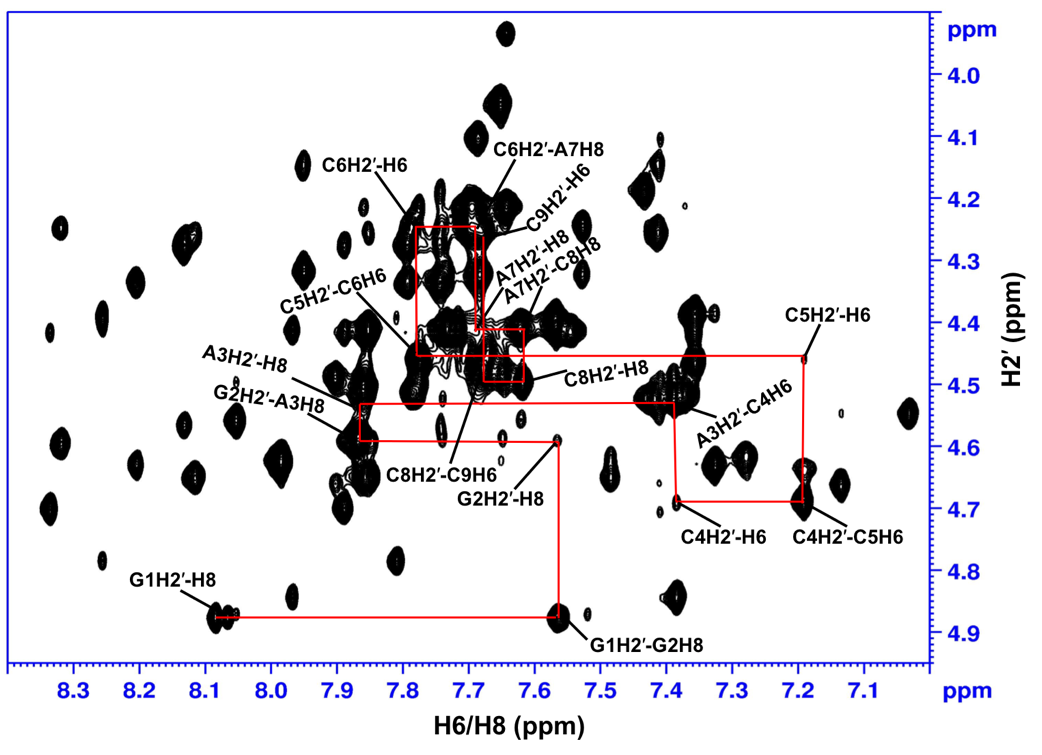


**Figure S8.** The H2′ to H6/H8 ‘helical walk’, annotated from G1 to C9, for A-paRNA-TL-tr, plotted on the 2D ^1^H-^1^H NOESY spectrum recorded at 25 °C with (H6/H8, H1′, H2′ but D3′, D4′, D5′/D5′′ and D5) ribose deuteration.


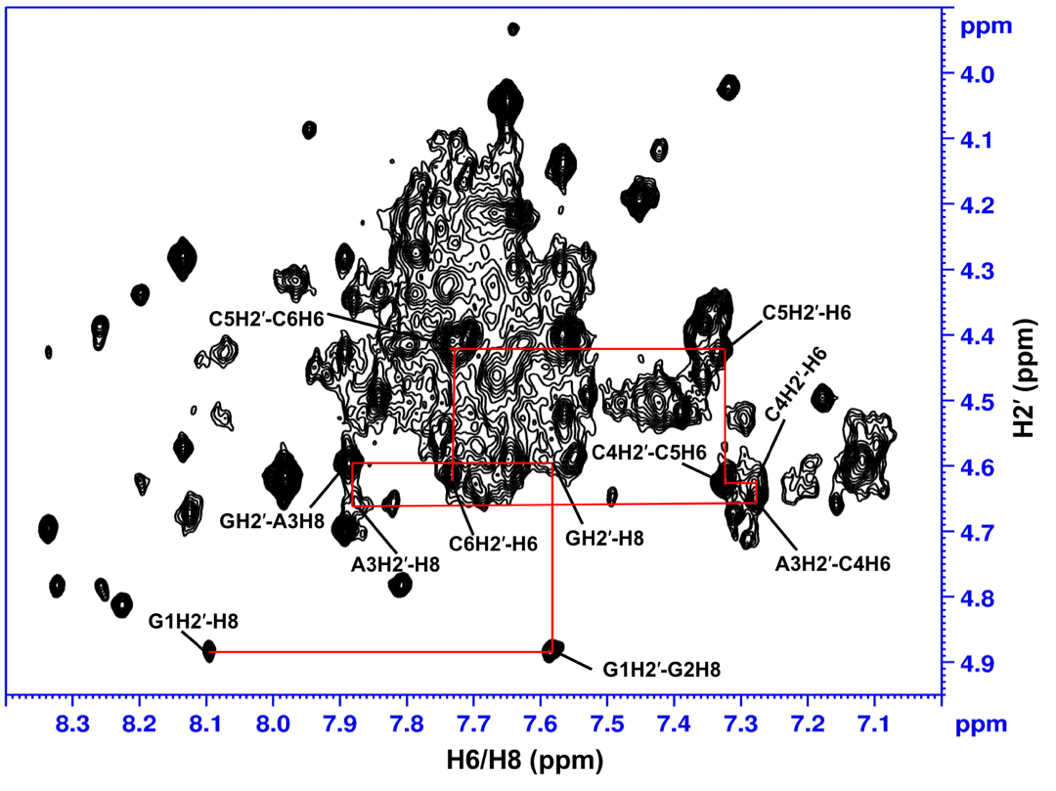


**Figure S9.** The H2′ to H6/H8 ‘helical walk’, annotated from G1 to C6, for A-paRNA-TL, plotted on the 2D ^1^H-^1^H NOESY spectrum recorded at 25 °C with (H6/H8, H1′, H2′ but D3′, D4′, D5′/D5′′ and D5) ribose deuteration. Even with extensive deuteration, overlap is considerable and again demonstrates the necessity of the divide-and-conquer approach.


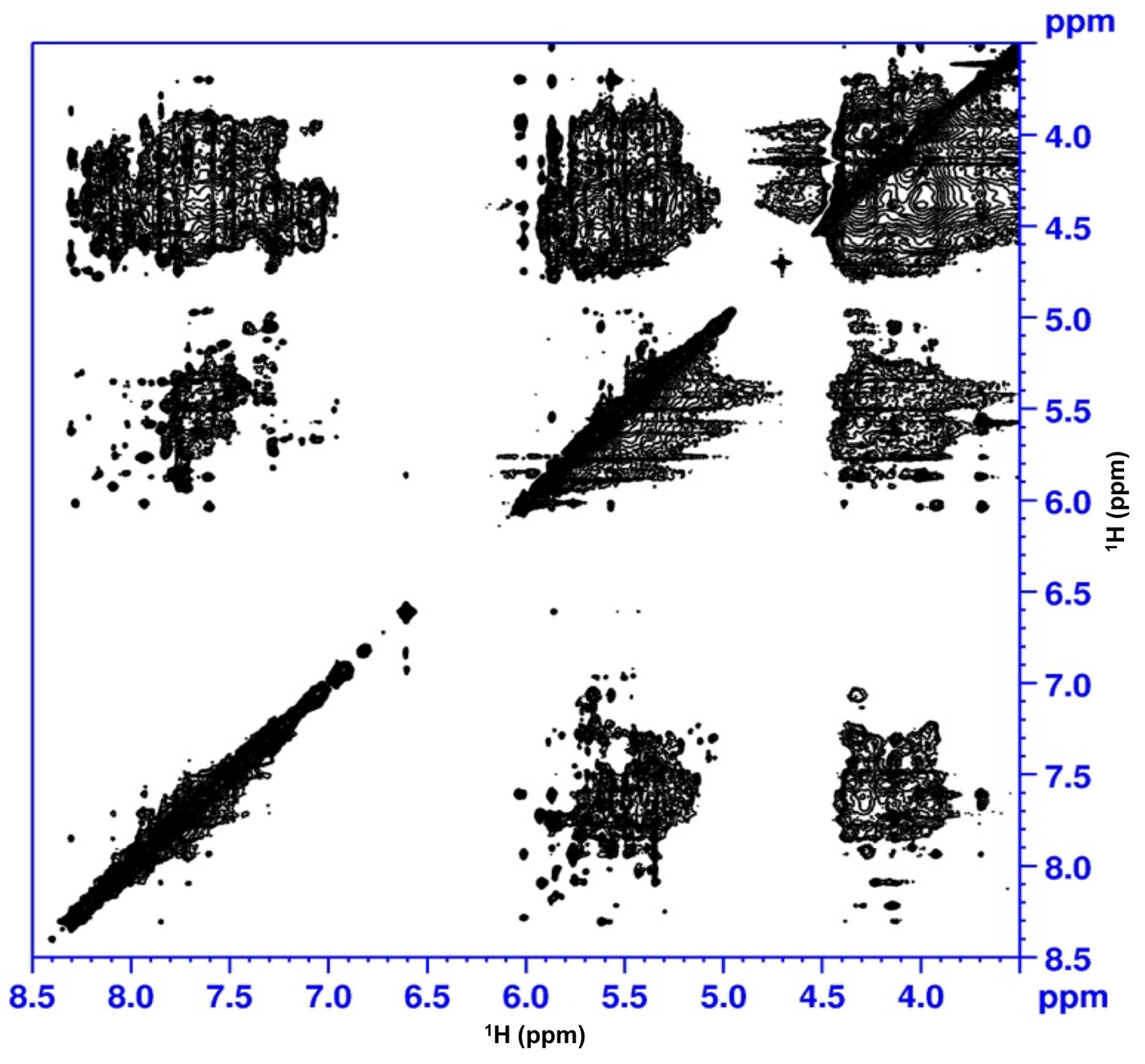


**Figure S10.** 2D ^1^H-^1^H NOESY spectrum (non-exchangeable protons) for A-paRNA-WT recorded at 25 °C. The broad and highly overlapped signals for the non-exchangeable protons are expected for an RNA of this size and would make structure determination impossible.

**
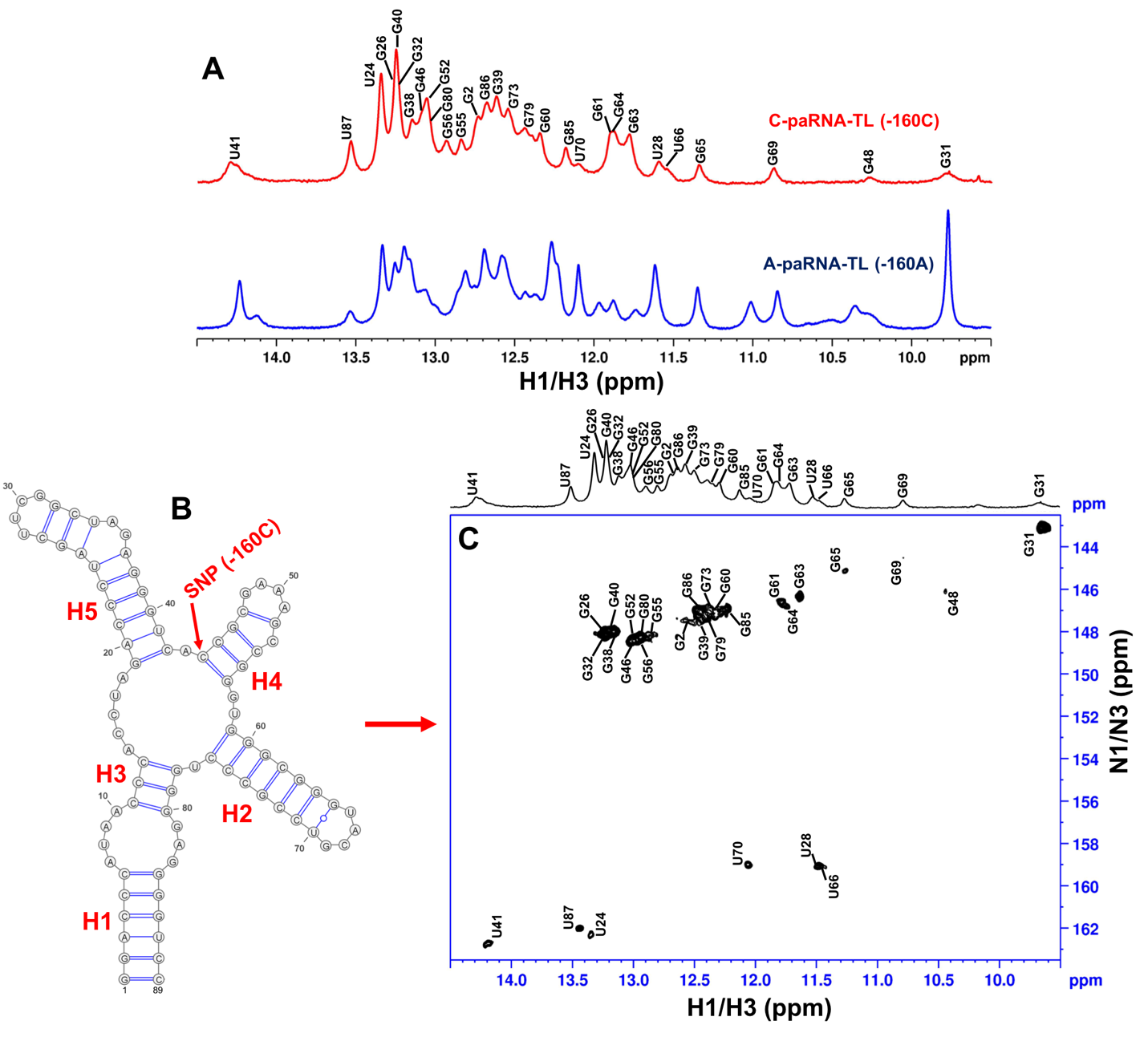
**

**Figure S11.** A and C variants differ substantially in their secondary structure. A) An overlay of the imino ^1^H NMR spectra for the A-paRNA-TL (-160A allele) and C-paRNA-TL (-160C allele) recorded at 15 °C. B) Secondary structure of C-paRNA-TL, as established from the NMR assignments (and fully consistent with the SHAPE analysis). C) 2D ^1^H-^15^N-HSQC spectrum of the same RNA construct recorded at 15 °C, with NMR assignments.


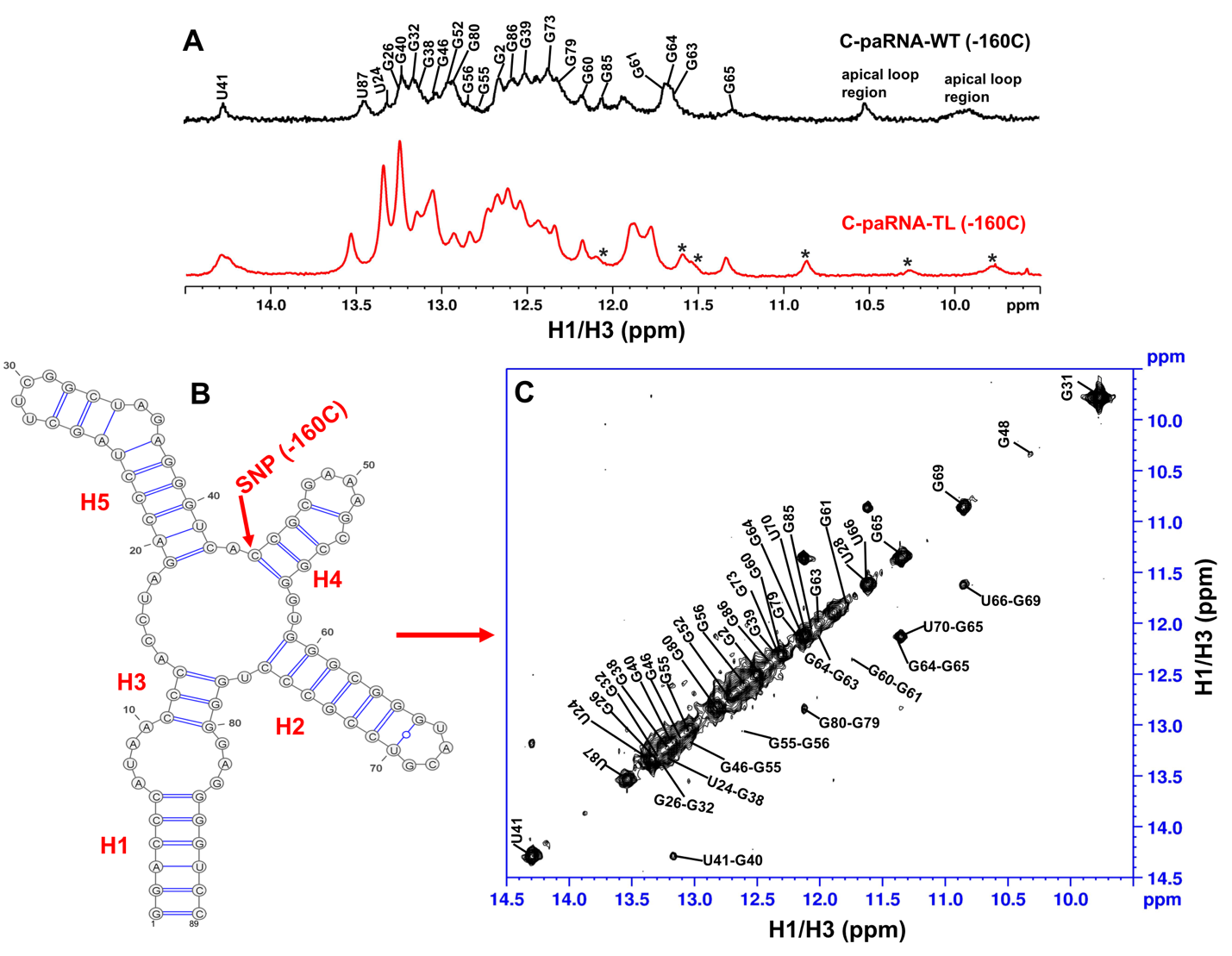


**Figure S12**. A) An overlay of the imino ^1^H NMR spectra for the C-paRNA-TL (-160C allele) and C-paRNA-WT (-160C allele) recorded at 15 °C. Comparison of the 1D imino ^1^H NMR spectra of the two RNAs, with assignments for the C-paRNA-TL, obtained as presented in the text, mapped onto the spectrum of the C-paRNA sequence (wild type loops). The spectra are very similar, indicative of conserved structures. Asterisks identify resonances originating from the UUCG, GAAA and UACG tetraloops, within the C-paRNA-TL construct; B) Secondary structure of C-paRNA-TL, as established from the NMR assignments (and fully consistent with the SHAPE analysis). C) 2D^1^H-^1^H NOESY spectrum of the same RNA construct at 15 °C, with NMR assignments.


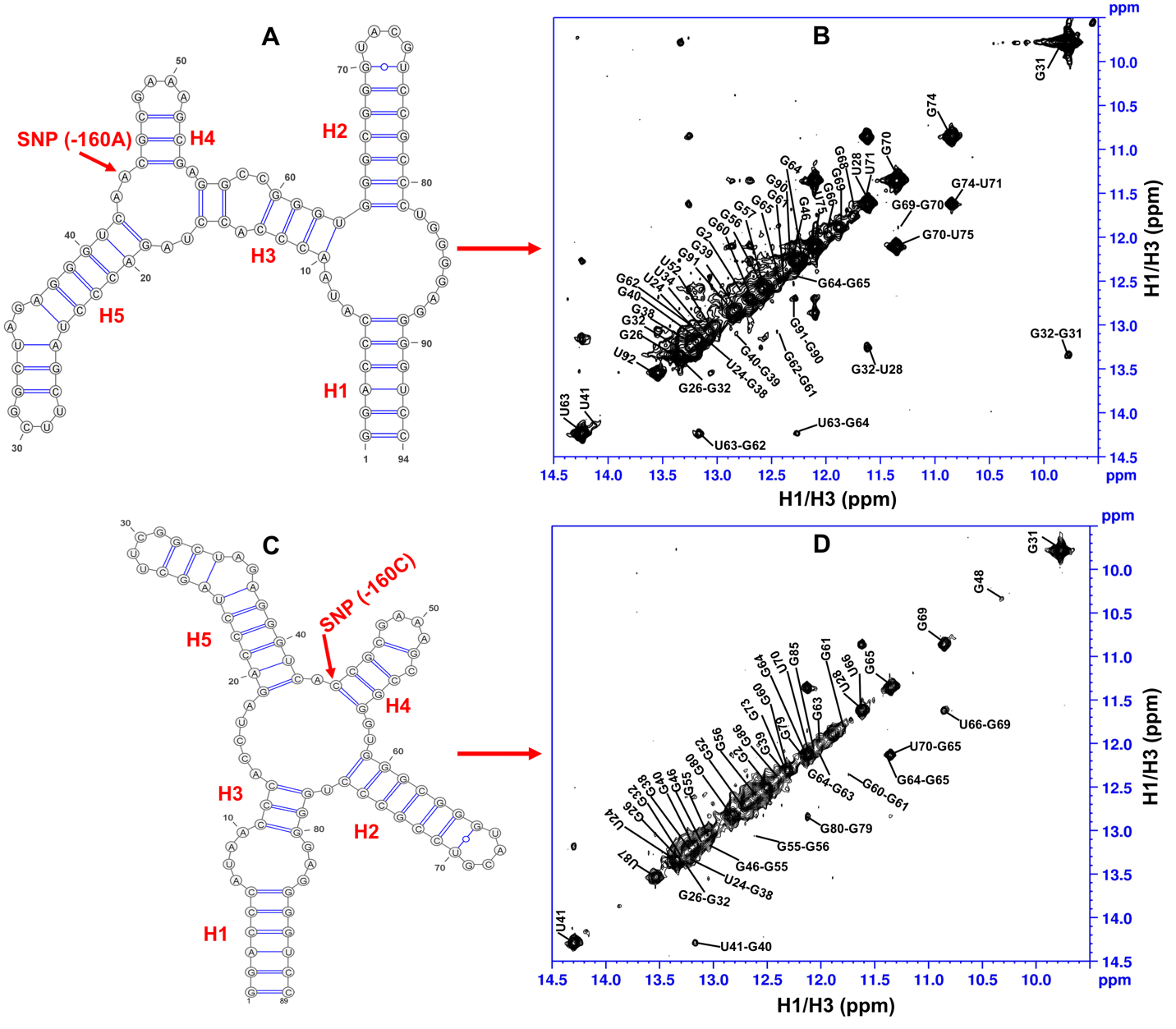


**Figure S13.** A) Secondary structure of A-paRNA-TL, as established by NMR. B) Imino region of the 2D^1^H-^1^H NOESY NMR spectrum of A-paRNA-TL recorded at 15 °C, with assignments that verify the secondary structure shown in A). C) Secondary structure of C-paRNA-TL, as established by NMR. D) Imino region of the 2D^1^H-^1^H NOESY NMR spectrum of C-paRNA-TLrecorded at 15 °C, with assignments that verify the secondary structure shown in C).


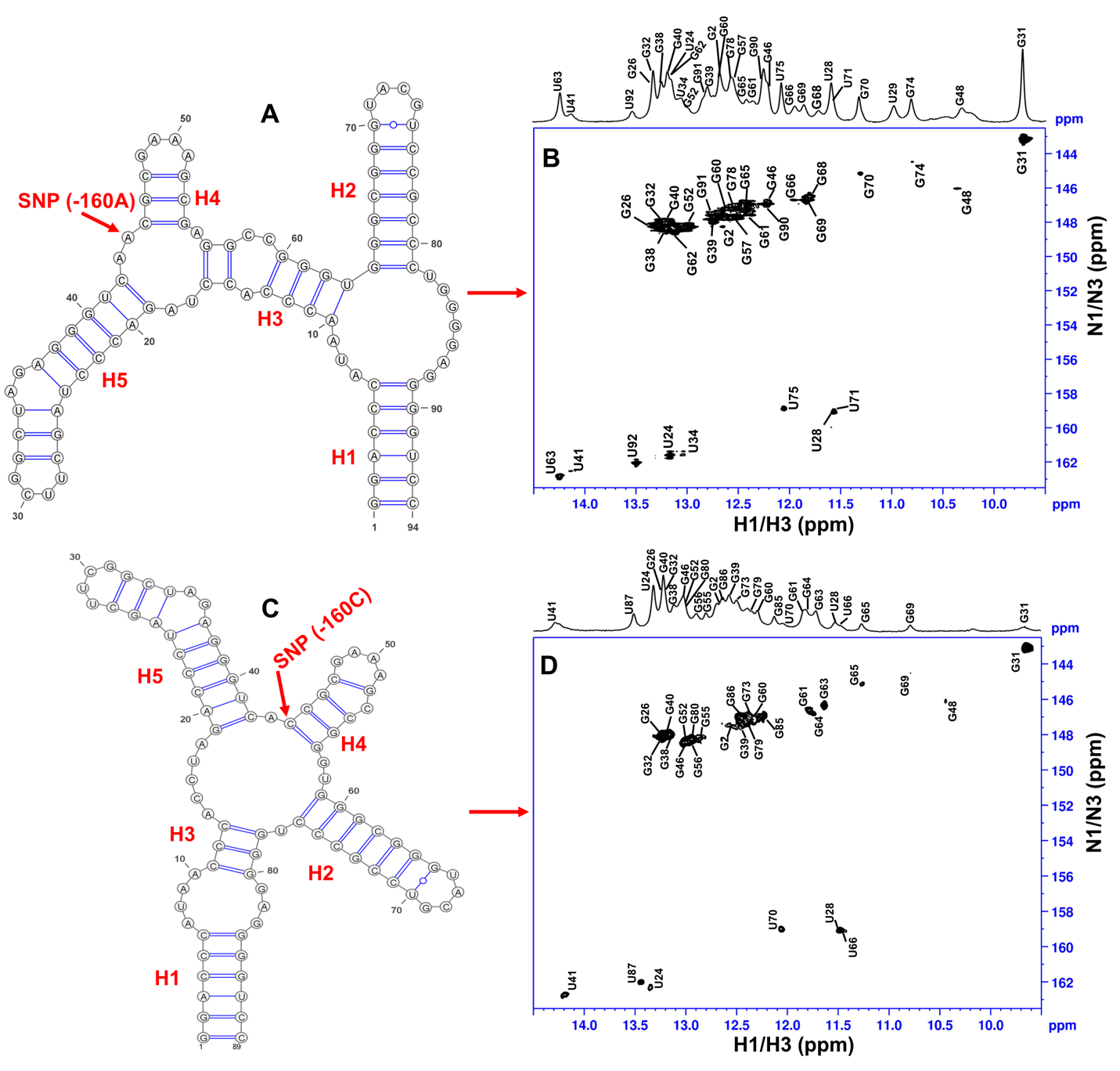


**Figure S14.** A) Secondary structure of A-paRNA-TL, as established by NMR. B) 2D ^1^H-^15^N HSQC spectra of the same RNA construct recorded at 15 °C, with NMR assignments that verify the secondary structure shown in A). C) Secondary structure of C-paRNA-TL, as established by NMR. D) 2D ^1^H-^15^N HSQC spectra of the same RNA construct recorded at 15 °C, with NMR assignments that verify the secondary structure shown in C).


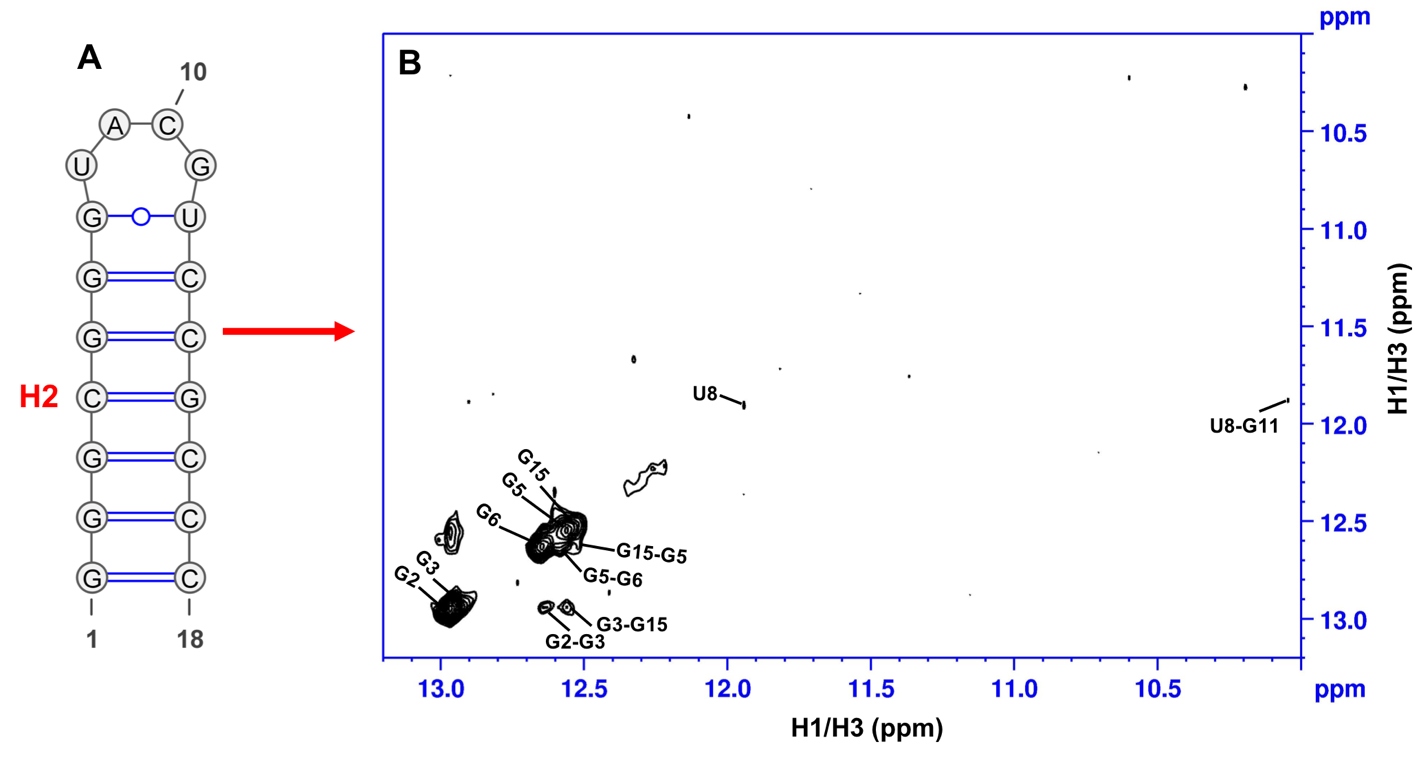


**Figure S15**. A) NMR-derived secondary structure of A-paRNA-2, corresponding to the isomiR-binding site, helix H2. B) Imino region of the 2D^1^H-^1^H NOESY NMR spectrum of A-paRNA-2 recorded at 15 °C, with assignments that verify the secondary structure shown in A).


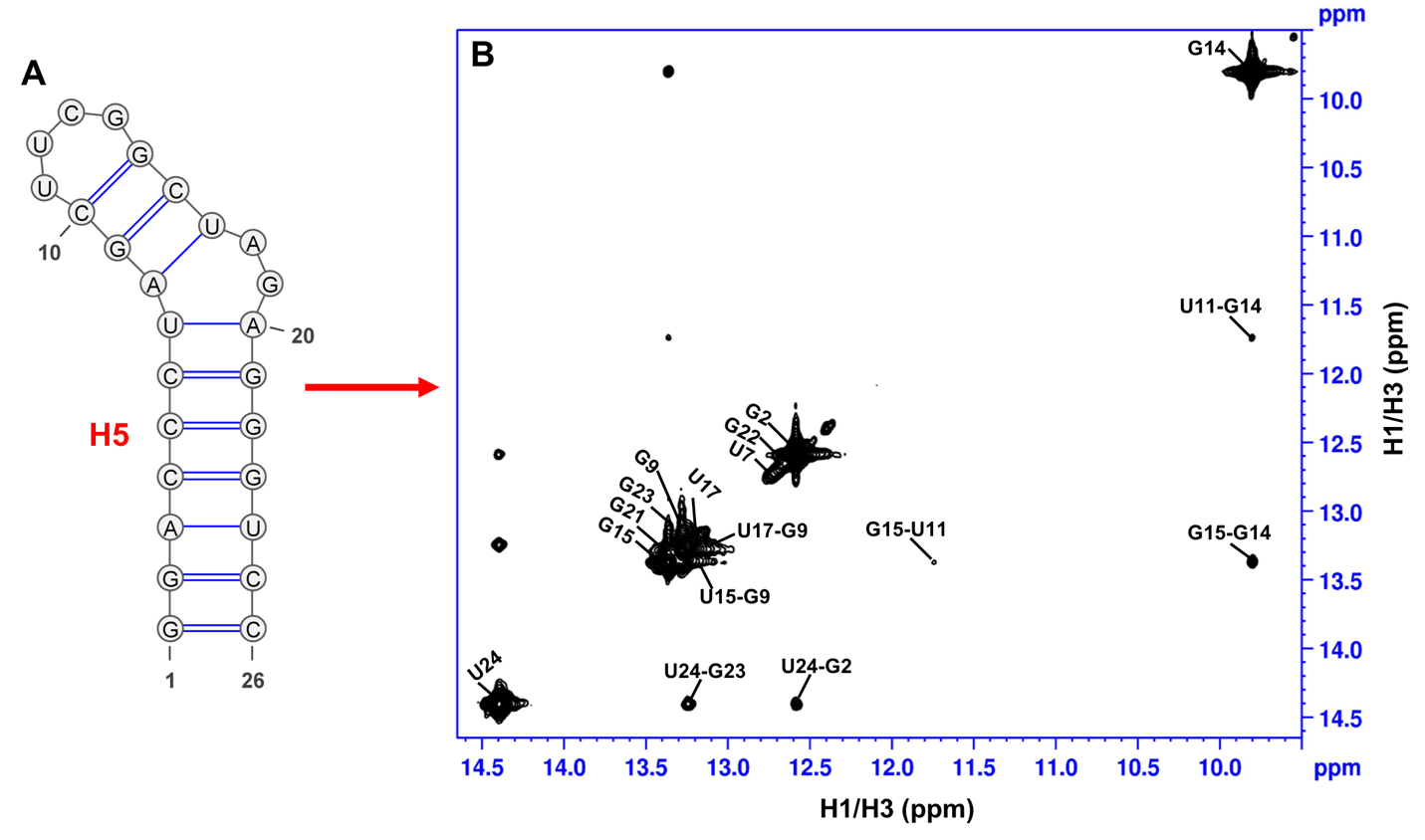


**Figure S16**. A) NMR-derived secondary structure of A-paRNA-TL-tr-2, corresponding to the large stem-loop emerging from the 3-way junction where the SNP is located, helix H5. B) Imino region of the 2D^1^H-^1^H NOESY NMR spectrum of A-paRNA-TL-tr-2 recorded at 15 °C, with assignments that verify the secondary structure shown in A).


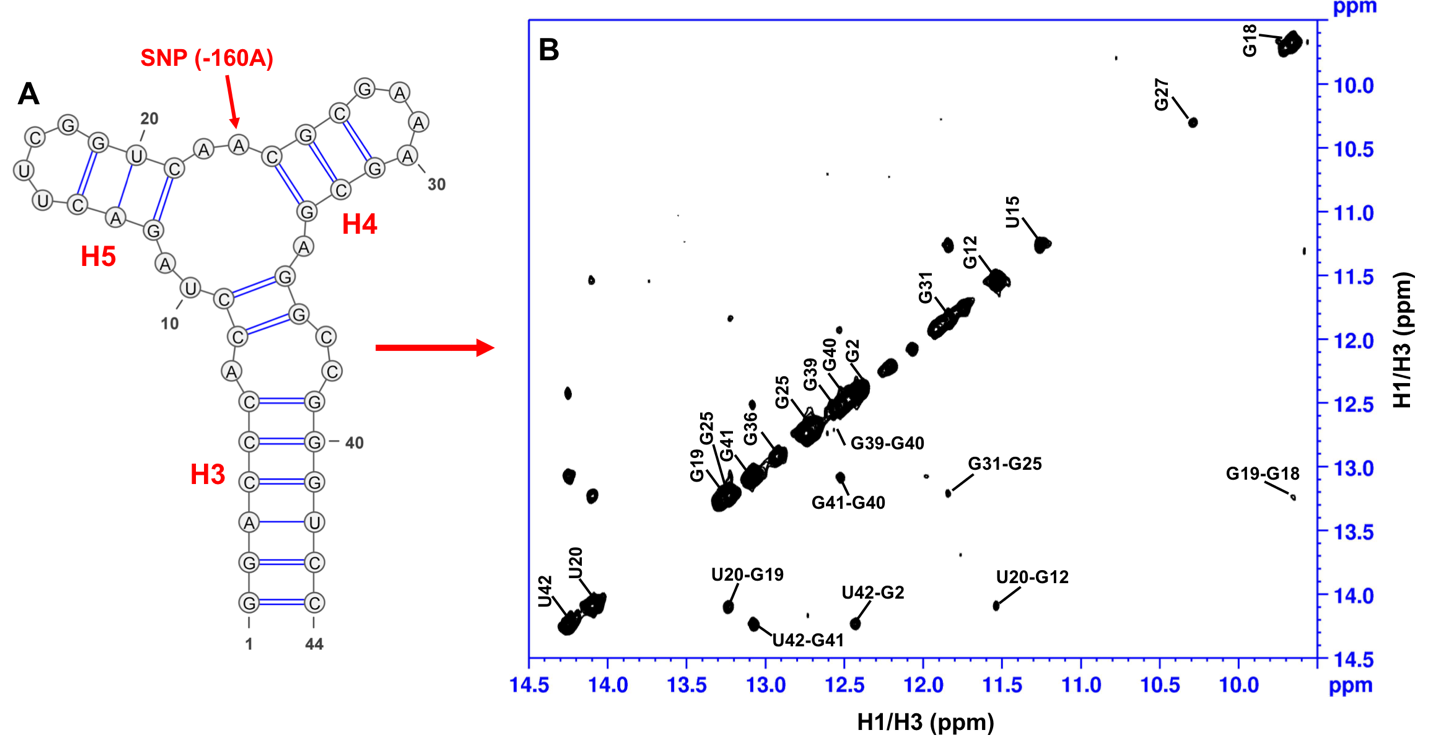


**Figure S17**. A) NMR-derived secondary structure of A-paRNA-TL-tr-1, which isolates the 3-way junction formed by helices H3, H4 and H5, where the SNP is located within a smaller RNA better amenable to high-resolution investigation. B) Imino region of the 2D^1^H-^1^H NOESY NMR spectrum of A-paRNA-TL-tr-1 recorded at 15 °C, with assignments that verify the secondary structure shown in A).


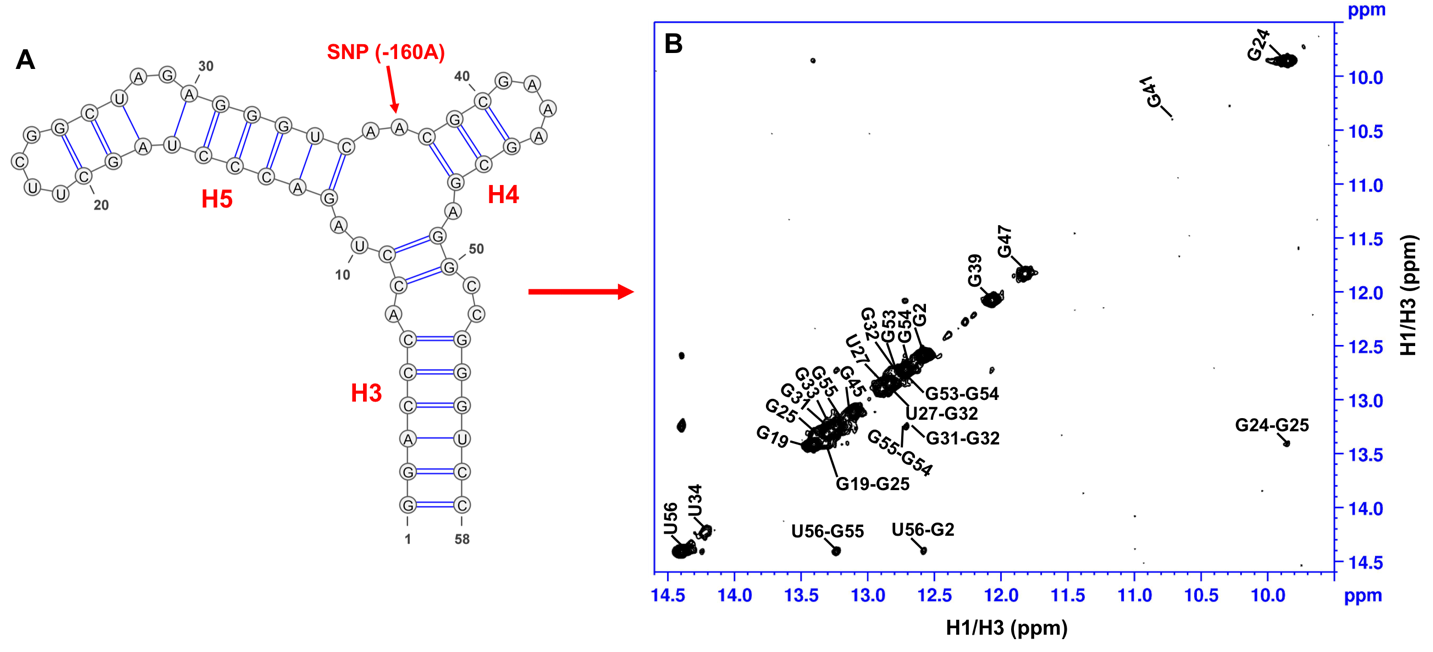


**Figure S18**. A) NMR-derived secondary structure of A-paRNA-TL-tr, that isolates the 3-way junction where the SNP is located. B) Imino region of the 2D^1^H-^1^H NOESY NMR spectrum of A-paRNA-TL-tr recorded at 15 °C, with assignments that verify the secondary structure shown in A).


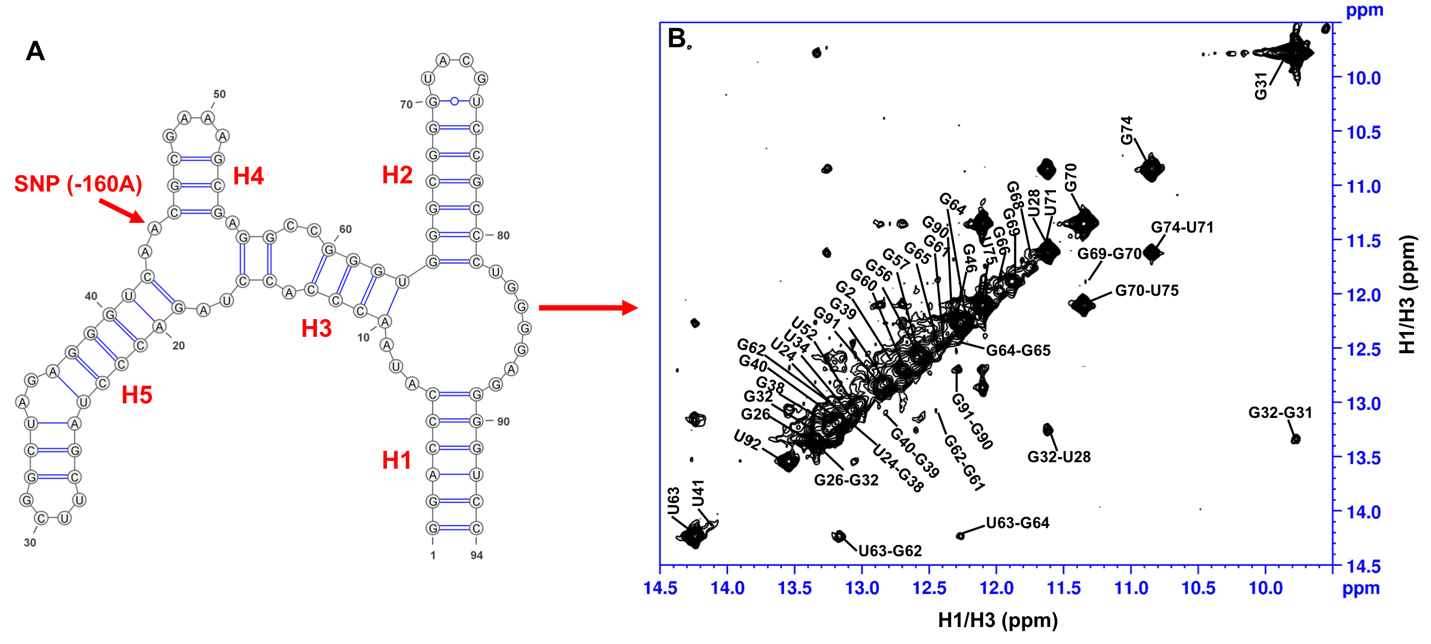


**Figure S19**. A) SHAPE-derived secondary structure of A-paRNA-TL, with tetraloops that stabilize the structure and reduce aggregation. B) Imino region of the 2D^1^H-^1^H NOESY NMR spectrum of A-paRNA-TL recorded at 15 °C, with assignments that verify the secondary structure, as shown in A), fully consistent with the SHAPE analysis.


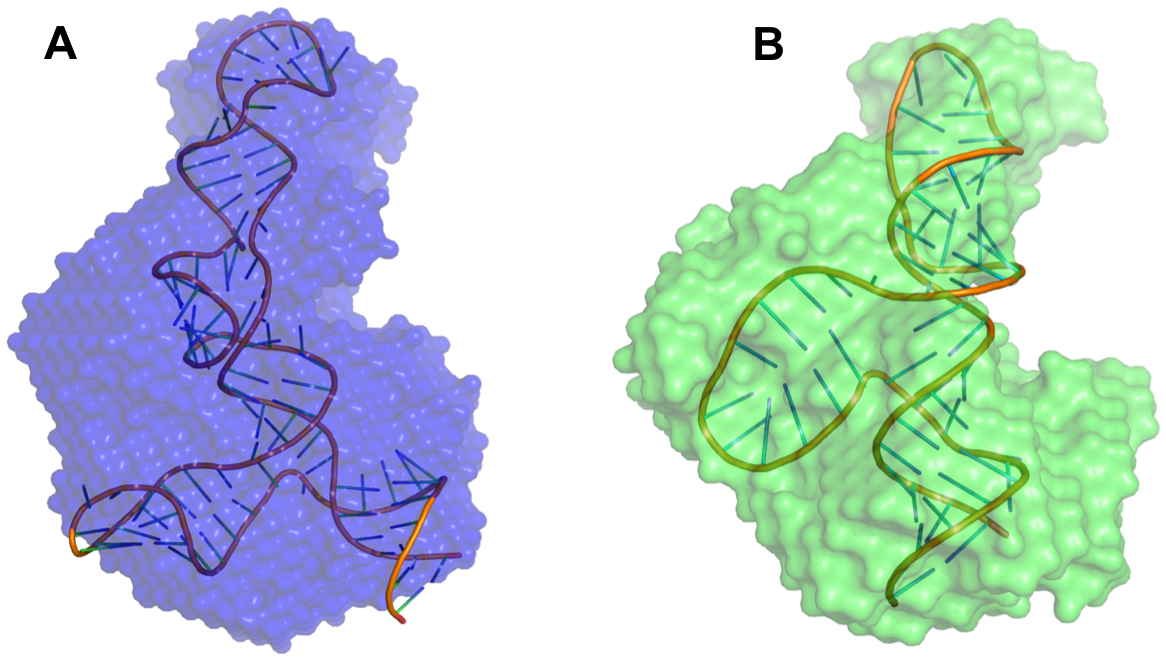


**Figure S20.** Comparison of the lowest energy NMR structures of A-paRNA-TL and A-paRNA-TL-tr with the corresponding SAXS envelopes shows good agreement between the two methods. A) The lowest energy structure of A-paRNA-TL in cartoon representation superposed on the SAXS model shown in blue; the A-paRNA-TL NMR structure fits within the corresponding SAXS envelope. B) The lowest energy structure of A-paRNA-TL-tr in cartoon representation superposed on the SAXS model shown in green; the A-paRNA-TL-tr structure shows good agreement with the SAXS envelope. The SAXS results were not used for NMR refinement.


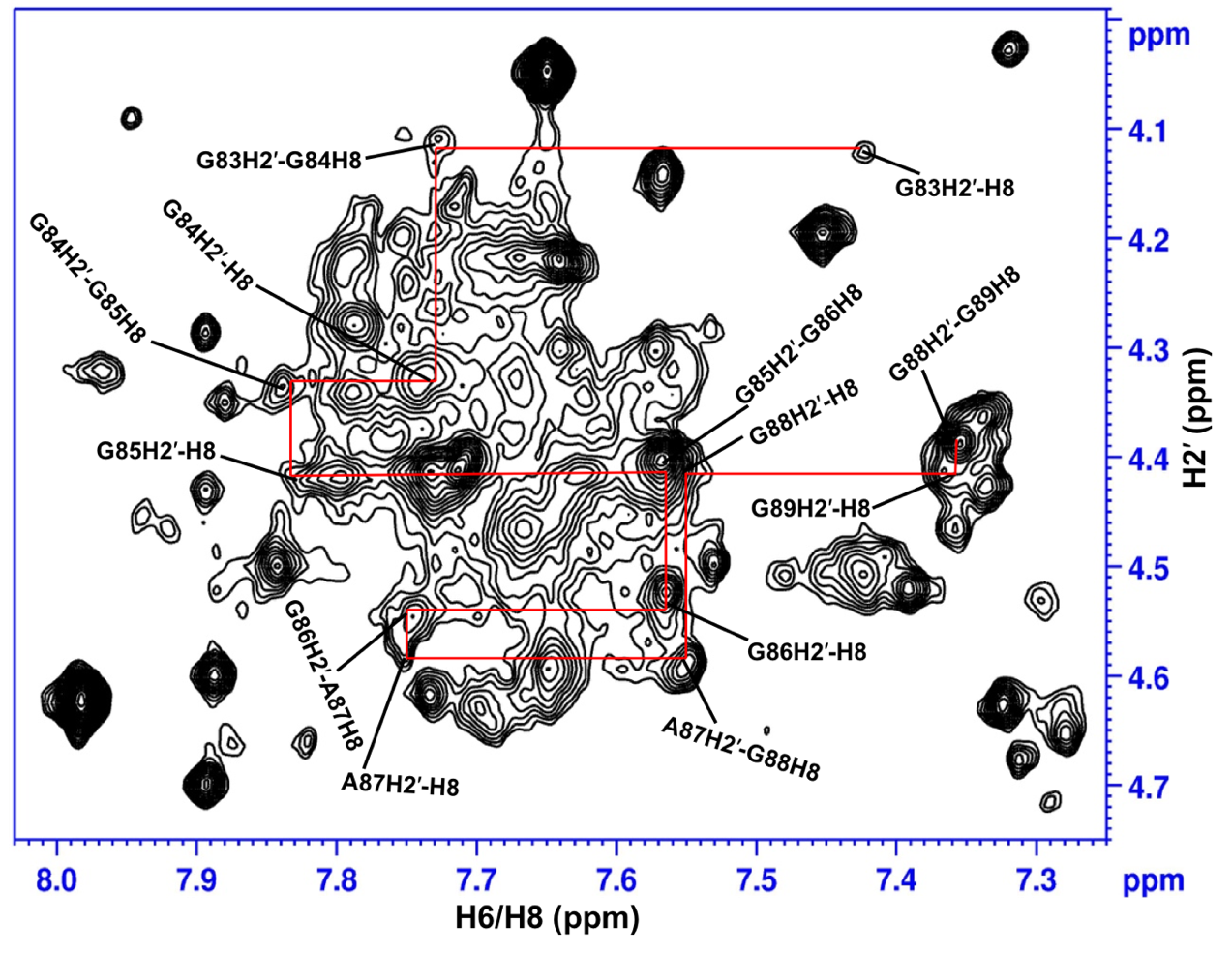


**Figure S21.** The H2′ to H6/H8 ‘helical walk’, labelled from G83 to G89, for A-paRNA-TL. The 2D ^1^H-^1^H NOESY spectrum was recorded at 25 °C with (H6/H8, H1′, H2′ but D3′, D4′, D5′/D5′′ and D5) ribose deuteration.


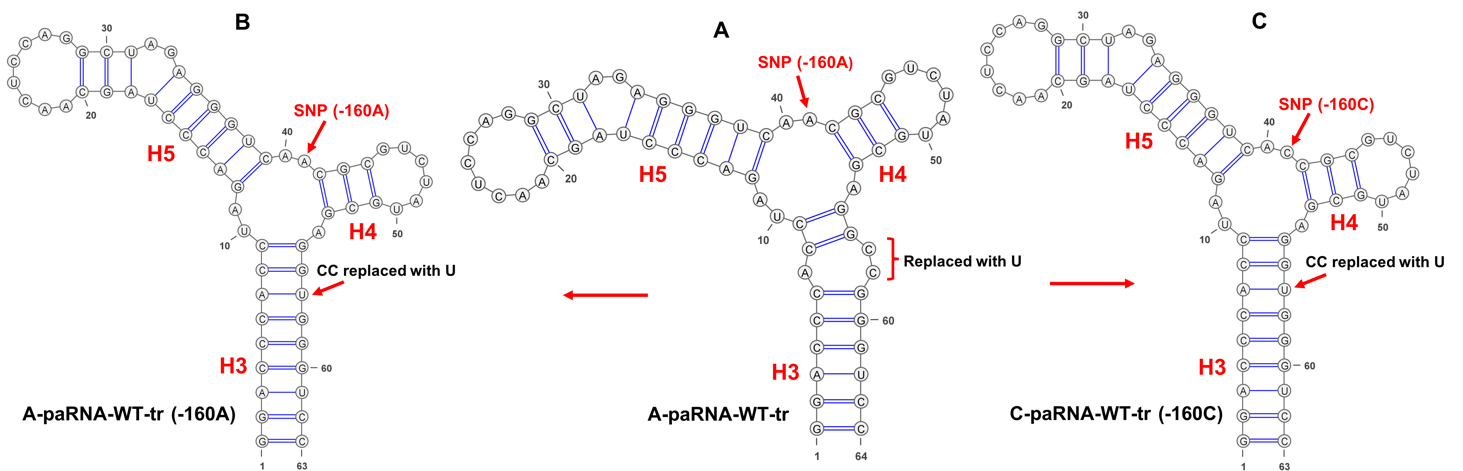


**Figure S22.** A) Secondary structure of A-paRNA-TL-tr. B) and C) are the secondary structures of the A and C-variants of the same RNA, but in these two constructs C58 and C59 (which are part of the internal loop in helix H3) are replaced with U, as shown with red arrows, to generate a perfectly paired helix H3. These constructs contain the natural loop sequences and not stabilizing tetraloops.


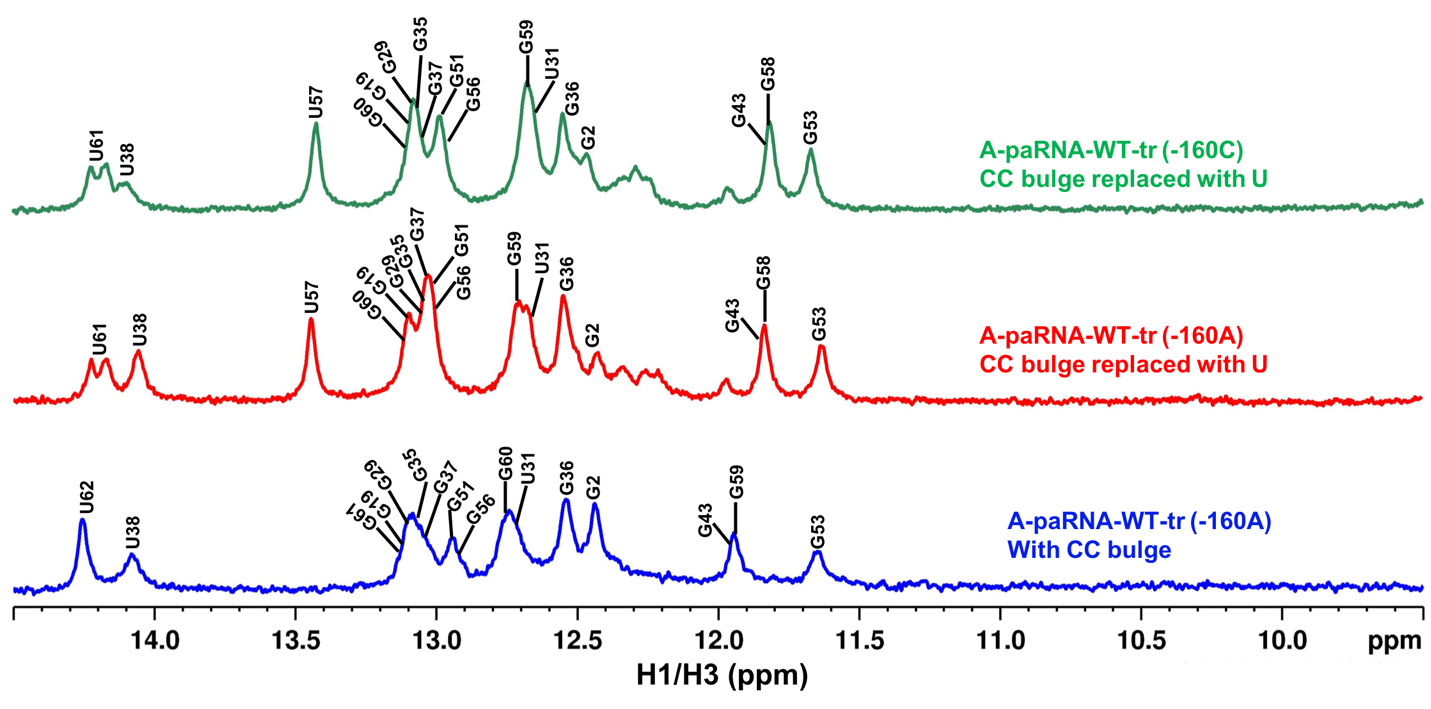
 **Figure S23.** Comparison of the 1D imino ^1^H NMR spectra recorded at 15 °C for the three RNAs of Fig. S22, corresponding to the helix H3, H4 and H5 portion of the paRNA that surround the SNP three-way junction. (Bottom) Spectrum for the construct with the A allele; (Center) Spectrum for the same RNA, but with C58C59 mutated to U to eliminate the internal loop and create a perfectly paired helix H3, resulting in a new AU base pair and, (Top) the same perfectly paired helix H3, but with the C allele at position -160 in the three-way junction. The three spectra are very similar, with the exception of the sharp resonance at about 13.5 ppm, corresponding to the new AU base pair created by the mutation. Once helix H3 is stabilized by replacing the internal loop with a perfectly paired helix, the A to C substitution in the three-way junction does not induce a conformational change.

**
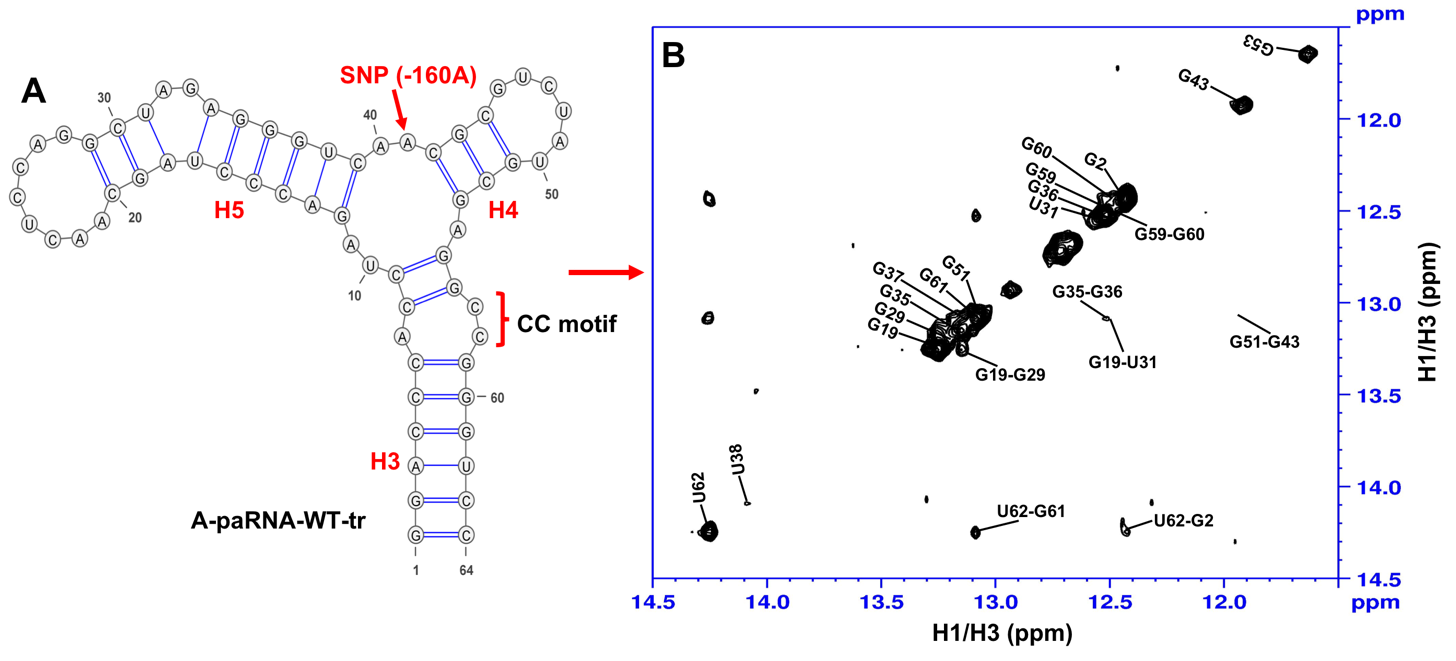
**

**Figure S24.** A) NMR-derived secondary structure of A-paRNA-WT-tr (-160A), that isolates the three-way junction where the SNP is located. B) Imino region of the 2D^1^H-^1^H NOESY NMR spectrum of A-paRNA-WT-tr (-160A) recorded at 15 °C, with assignments that verify the secondary structure shown.

**
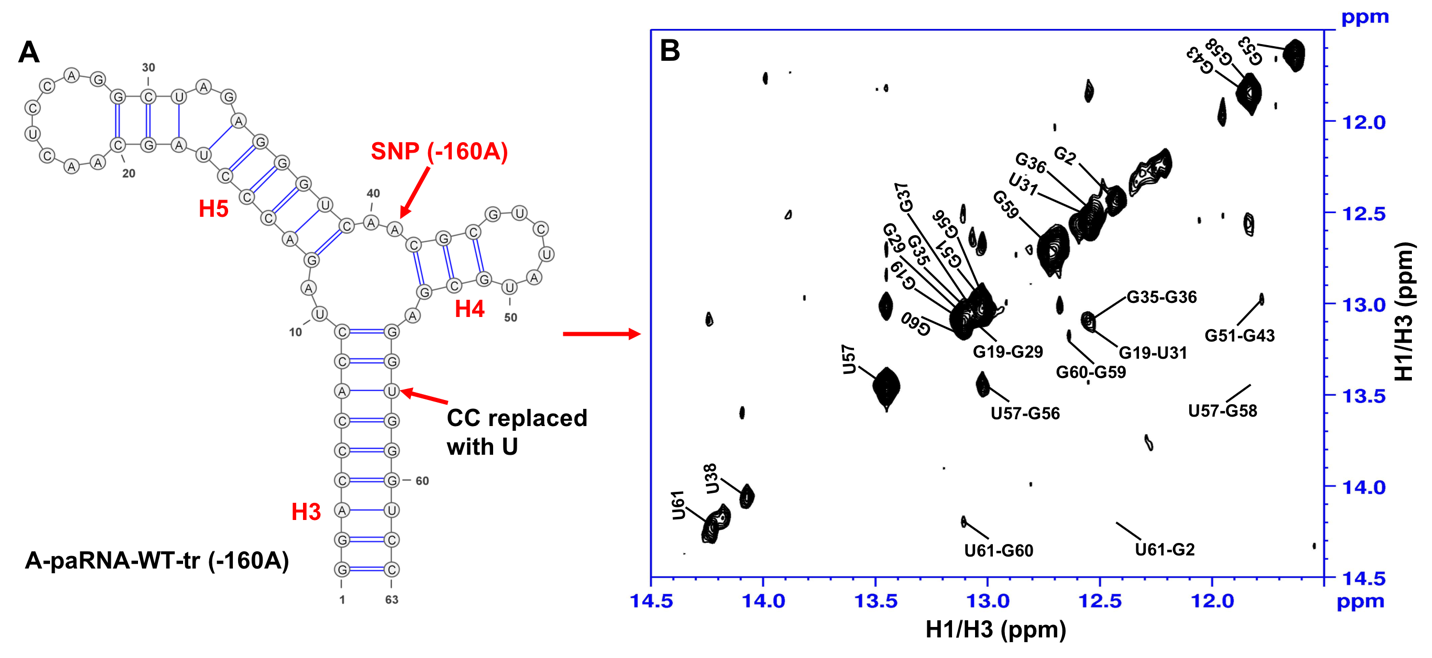
**

**Figure S25.** A) NMR-derived secondary structure of A-paRNA-WT-tr (-160A), that isolates the three-way junction where the SNP is located. In this construct, the CC motif, which is part of the internal loop in helix H3has been replaced by a U (indicated by the red arrow), to generate a perfectly paired helix H3. B) Imino region of the 2D^1^H-^1^H NOESY NMR spectrum of A-paRNA-WT-tr (-160A) recorded at 15 °C, with assignments that verify the secondary structure shown in A).

**
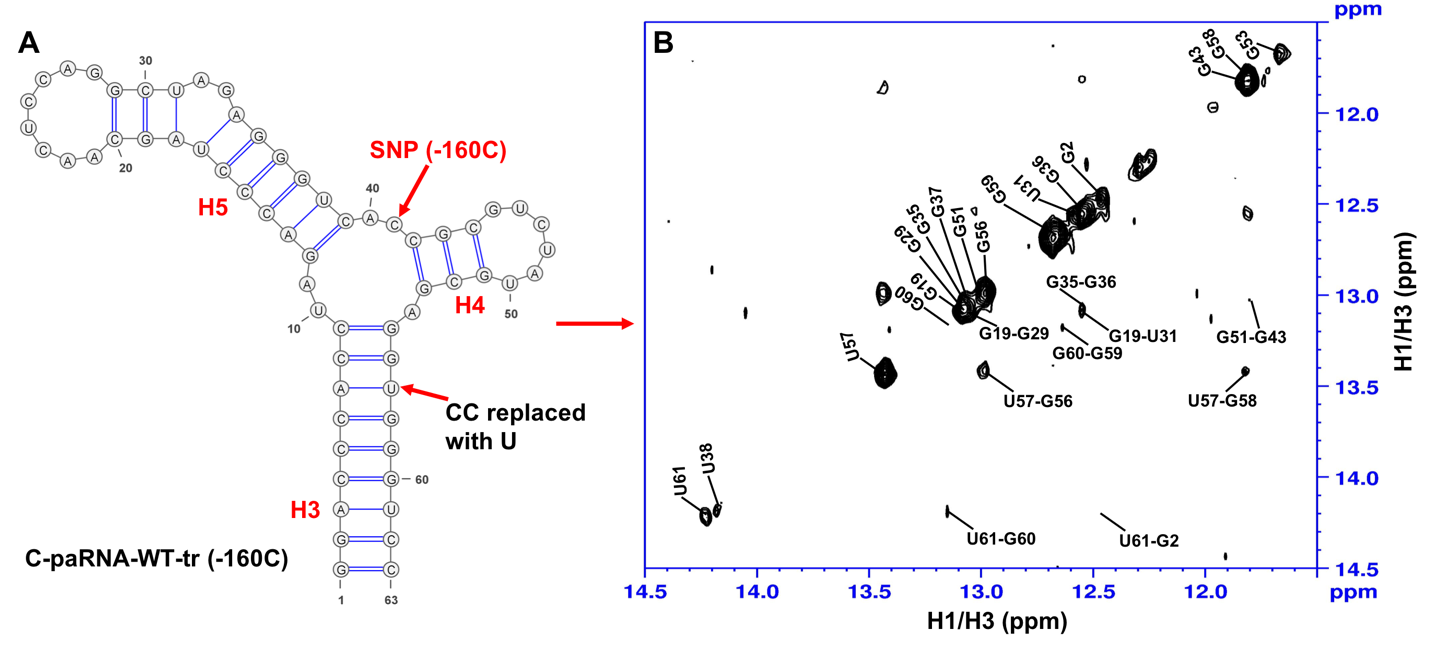
**

**Figure S26.** A) NMR-derived secondary structure of C-paRNA-WT-tr (-160C), that isolates the three-way junction where the SNP is located. In this construct, the CC motif which is part of the internal loop in helix H3, has been replaced by a U (indicated by the red arrow), to generate a perfectly paired helix H3. B) Imino region of the 2D^1^H-^1^H NOESY NMR spectrum of C-paRNA-WT-tr (-160C) recorded at 15 °C, with assignments that verify the secondary structure shown in A).

**
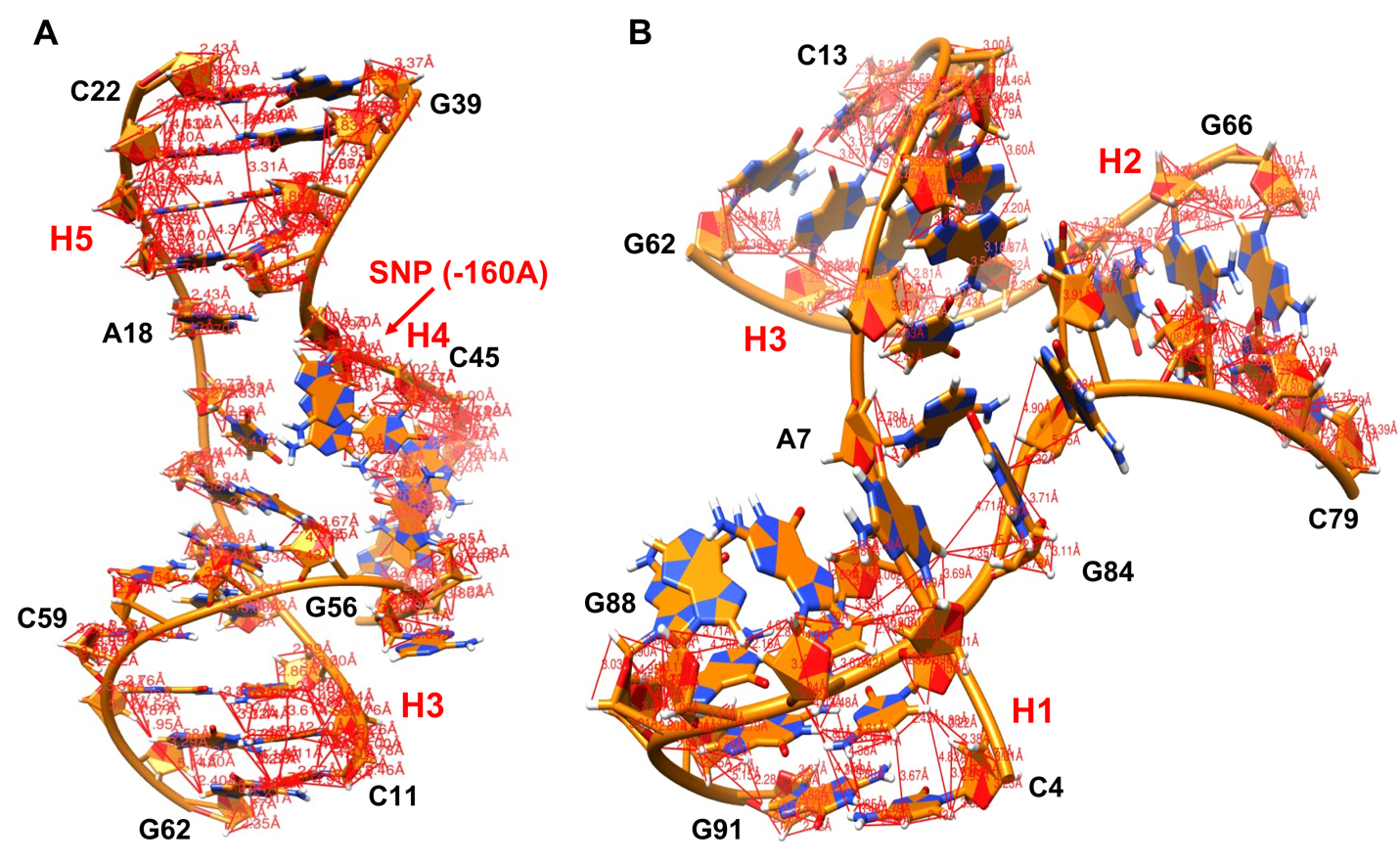
**

**Figure S27.** NMR distance restraints that provide information about the arrangement of the three-way junction helical stems, mapped onto the 3D NMR structure of A-paRNA-TL. A) Close up view of the three-way junction formed by helices H3, H4 and H5, where the SNP (-160A) is located. B) Close up view of the three-way junction from which the isomiR binding site emanates, formed by helices H1, H2 and H3. This image shows the large number of NOE-derived distance restraints per nucleotide by using the divide and conquer approach for A-paRNA-TL, as needed for high quality 3D NMR structure determination.
